# Structural determinants and regulation of spontaneous activity in GABA_A_ receptors

**DOI:** 10.1038/s41467-021-25633-0

**Published:** 2021-09-15

**Authors:** Craig A. Sexton, Reka Penzinger, Martin Mortensen, Damian P. Bright, Trevor G. Smart

**Affiliations:** Department of Neuroscience, Physiology & Pharmacology, UCL, London, UK

**Keywords:** Ligand-gated ion channels, Patch clamp, Single-channel recording, Neuroscience

## Abstract

GABA_A_ receptors are vital for controlling neuronal excitability and can display significant levels of constitutive activity that contributes to tonic inhibition. However, the mechanisms underlying spontaneity are poorly understood. Here we demonstrate a strict requirement for β3 subunit incorporation into receptors for spontaneous gating, facilitated by α4, α6 and δ subunits. The crucial molecular determinant involves four amino acids (GKER) in the β3 subunit’s extracellular domain, which interacts with adjacent receptor subunits to promote transition to activated, open channel conformations. Spontaneous activity is further regulated by β3 subunit phosphorylation and by allosteric modulators including neurosteroids and benzodiazepines. Promoting spontaneous activity reduced neuronal excitability, indicating that spontaneous currents will alter neural network activity. This study demonstrates how regional diversity in GABA_A_ receptor isoform, protein kinase activity, and neurosteroid levels, can impact on tonic inhibition through the modulation of spontaneous GABA_A_ receptor gating.

## Abstract (web summary)

GABA_A_ receptors (GABA_A_Rs) cause inhibition in the brain by functioning as heteropentamers formed from multiple subunit types. Here, the authors demonstrate that receptors incorporating β3 subunits can spontaneously gate, which is modulated by protein kinases and neurosteroids to affect tonic inhibition.

## Introduction

Inhibition in the brain is predominantly provided by the neurotransmitter γ-aminobutyric acid (GABA) activating GABA_A_ receptors (GABA_A_Rs). These heteropentameric receptors are formed from an array of subunits (α1-6, β1-3, γ1-3, δ, ε, π, θ and ρ1-3), producing receptors with diverse functional and pharmacological properties^1,2^. Despite the potential for significant diversity, limited isoforms are expressed in vivo due to the preferential assembly of subunits^3^. The most common subunit configuration consists of two α, two β and a single γ or δ subunit in a clockwise α-β-α-β-γ/δ arrangement, when viewed extracellularly^4,5^. Synaptic receptors typically comprise α1-3, β and γ subunits, while extrasynaptic receptors contain α4/6, β and δ subunits^6^. Receptors composed of α5, β and γ subunits localise to both synaptic and extrasynaptic areas^7,8^.

Extrasynaptic receptors typically exhibit higher sensitivities to GABA and less desensitisation than their synaptic counterparts, permitting mediation of tonic inhibition through persistent activation by ambient GABA^6,9,10^. Previous studies in hippocampal neurons have also reported a GABA-independent, GABA_A_R-mediated, component to the tonic current^11–13^. This accords with previous demonstrations of spontaneity in recombinant GABA_A_R isoforms, including: β1/3 homomers^14,15^, α1β1 diheteromers^16^, ε-containing receptors^17^ and the more widely expressed α1β1γ2 and α4β3δ receptors^16,18^. This spontaneous activity is manifest at the single-channel level by brief, infrequent openings in the absence of agonist, resulting in a persistent holding current that contributes towards tonic inhibition^13,18–20^. The effects of tonic inhibition on neuronal excitability have been extensively studied. In addition to membrane hyperpolarisation produced by GABA_A_R activation in most mature neurons, continuously active receptors reduce membrane resistance, decreasing both the magnitude, duration and length of travel for membrane voltage changes occurring in response to transmembrane ionic current^21,22^. This shunting inhibition modulates the propagation and integration of excitatory inputs, controlling neuronal input–output relationships^21,23^. By contributing to tonic inhibition, spontaneously active GABA_A_Rs are predicted to have important consequences for neuronal computation and network activity^13^.

In this study we have identified GABA_A_R isoforms that display a propensity for spontaneous gating. Notably, the β3 subunit is essential for enabling heteromers to gate spontaneously, and this depends on key residues within the extracellular domain that can interact with adjacent subunits to stabilise an agonist-independent open channel conformation. These spontaneous currents are regulated by endogenous modulators, such as protein kinases and neurosteroids, and by other ligands. Our results reveal that modulation of spontaneous currents regulates neuronal excitability, representing an important physiological mechanism through which tonic inhibition can be dynamically controlled.

## Results

### β3 subunits are required for GABA_A_Rs to spontaneously gate

**Fig. 1:**
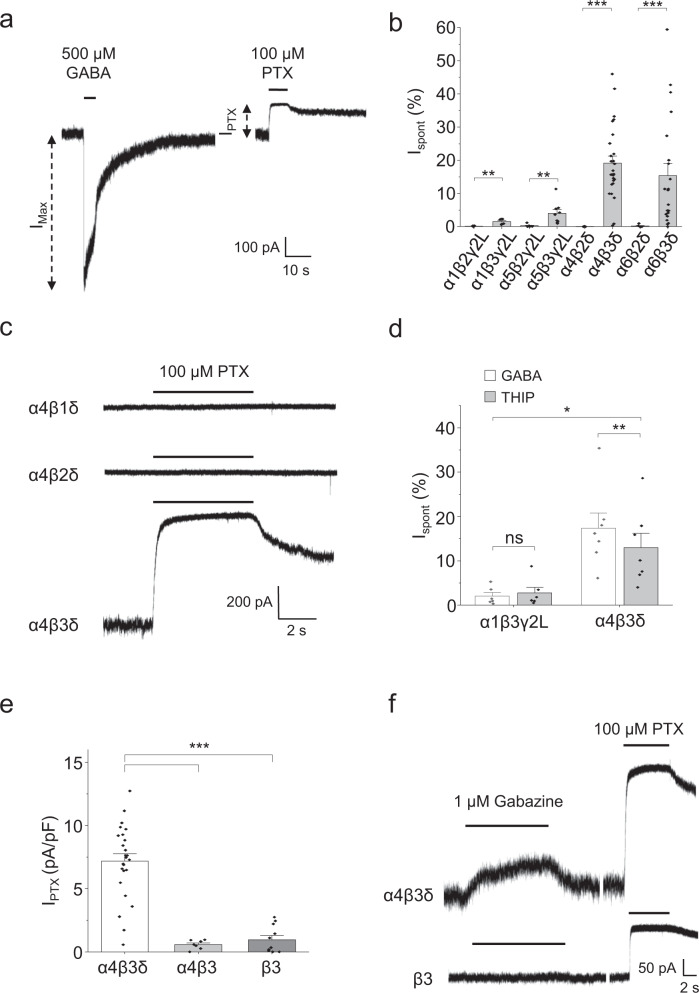
β3 subunits are required for heteromeric GABA_A_R spontaneous activity. **a** Determining spontaneous receptor activity in HEK cells expressing α4β3δ. I_Max_ is the current evoked by a saturating concentration of agonist (GABA, 500 µM), while I_PTX_ is the reduced holding current caused by a saturating concentration of PTX (100 µM) and is equivalent to the current caused by spontaneous GABA_A_R activity. These parameters were used to calculate I_spont_ (see Methods). **b** Bar graph of I_spont_ for a variety of receptor isoforms expressed in HEK cells (α1β2γ2L/α1β3γ2L: *n* (cells) = 7/6, **P = 0.0093, two-sided unpaired *t*-test; α5β2γ2L/α5β3γ2L: *n* = 7/9, ***P = 0.00035, two-sided Mann–Whitney test; α4β2δ/α4β3δ: *n* = 6/27, ***P = 1.8E-6, two-sided Mann–Whitney test; α6β2δ/α6β3δ: *n* = 7/21, ***P = 3E-5, two-sided Mann–Whitney test). **c** Representative spontaneous currents for α4β1δ, α4β2δ and α4β3δ receptors, determined using PTX (100 μM). **d** Using the ‘super agonist’ THIP (3 mM) to calculate I_spont_ of α4β3δ receptors (α1β3γ2L: *n* = 6, *P* = 0.26 two-sided paired *t*-test; α4β3δ: *n* = 7; **P = 0.0090 two-sided paired *t*-test. Between-receptor comparison: *P = 0.014, two-sided unpaired *t*-test). **e** HEK cells transfected with three (α4, β3 and δ), two (α4 and β3) or one (β3) cDNA to provide preferential conditions for the formation of triheteromeric, diheteromeric and homomeric receptors, respectively. Measurement of I_PTX_ was normalised to cell capacitance. Triheteromers displayed significantly larger current densities than diheteromers (***P = 0.00034) and homomers (***P = 2.8E-5). Current densities for diheteromers and homomers did not differ (*P* = 1; Kruskall–Wallis with Dunn’s post-hoc test; α4β3δ: *n* = 25; α4β3: *n* = 7; β3: *n* = 11). **f** The orthosteric antagonist gabazine (1 μM) exhibits inverse agonist properties at triheteromeric (top), but not homomeric, receptors. Data are presented as mean values ± SEM. *P < 0.05; **P < 0.01; ***P < 0.001; ns no significance. Source data are provided as a Source Data file for Fig. 1.

Common isoforms of the GABA_A_R were initially screened for spontaneous activity by recording from HEK cells expressing discrete synaptic- (α1β2/3γ2L) and extrasynaptic-type receptors (α4/6β2/3δ), and those capable of expression in both domains (α5β2/3γ2L). Spontaneous activity was quantified for each receptor subtype by sequentially applying saturating concentrations of GABA (500 µM) and picrotoxin (PTX, 100 µM) to determine the maximum agonist-activated (I_Max_) and picrotoxin-sensitive (I_PTX_) currents, respectively (Fig. 1a). PTX is a use-dependent allosteric antagonist at GABA_A_Rs and will inhibit constitutively active receptors. The I_PTX_ was then normalised (%) to the total GABA_A_R current (I_Max_ + I_PTX_) to estimate the level of spontaneous current (I_spont_). The data indicated that only receptors containing the β3 subunit displayed a spontaneous current. Exchanging β3 for β2 eliminated spontaneous gating, independent of the identity of co-assembled α, γ2 and δ subunits (Fig. 1b; Supplementary Fig. 1a; Supplementary Table 1). Spontaneous activity was also absent for receptors containing the β1 subunit co-assembled with α4 and δ (Fig. 1c; Supplementary Table 1).

Other subunits do affect spontaneity, notably δ-containing receptors support significantly higher levels of spontaneous current than γ-containing counterparts (Supplementary Fig. 1a). However, comparing normalised I_spont_ between these receptor subtypes is potentially confounded by the partial agonist behaviour of GABA at δ-containing receptors^24^. To account for this, we used the GABA_A_R agonist THIP (4,5,6,7-tetrahydroisoxazolo[5,4-c]pyridin-3-ol) to maximally activate δ-containing receptors^24^. As expected, I_Max_ for α4β3δ receptors was larger when using THIP (3 mM) compared to GABA, resulting in a small reduction of I_spont_ (Fig. 1d). Nevertheless, α4β3δ receptors still exhibited significantly larger I_spont_ than α1β3γ2L receptors when normalised using maximal currents evoked by full agonists. Therefore, even accounting for differences in agonist efficacies, spontaneous activity of extrasynaptic-type receptors is greater than for synaptic-type isoforms. Given that these extrasynaptic δ-GABA_A_Rs (α4β3δ and α6β3δ) are important for generating tonic current in various brain regions^22^, this suggests that tonic inhibition in the brain may represent a combination of GABA-dependent and -independent receptor activity.

Cells transfected with the three subunit cDNAs, α4, β3 and δ, could theoretically also express sub-populations of homomeric β3 and diheteromeric α4β3 GABA_A_Rs, along with triheteromeric α4β3δ receptors. To assess whether these two sub-populations contributed to the spontaneous current, especially given that homomeric β3 receptors are constitutively active^14^, we measured spontaneous currents generated by β3 and α4β3 receptors expressed separately to determine if these assemblies represent significant populations in cells expressing α4β3δ receptors.

Transfection with all three (α4, β3 and δ), two (α4 and β3) or just a single (β3) cDNA showed that only cells expressing all three subunits exhibited significant spontaneous currents (Fig. 1e, Supplementary Fig. 1b). Hence, even under conditions ideal for the assembly of α4β3 diheteromers and β3 homomers, these receptors showed little spontaneous activity, despite appreciable levels of expression (α4β3: 237 ± 95 pA activated by 500 µM GABA, *n* = 7; β3: 220 ± 94 pA evoked by 100 µM pentobarbital, *n* = 11). Pharmacological tools were also employed to assess the relative expression of these receptor sub-populations in α4β3δ-transfected cells. The competitive GABA_A_R antagonist gabazine (1 µM) behaved as an inverse agonist for cells expressing α4, β3 and δ receptors, but not with β3 alone (Fig. 1f), suggesting most receptors possessed an orthosteric binding site (e.g. α4β3, α4β3δ) contrasting with β3 homomers. To discriminate between α4β3 and α4β3δ receptors, we used Zn^2+^, which inhibits diheteromeric αβ receptors with significantly higher potency (nM) compared to triheteromeric αβδ receptors (μM)^25^. Application of 10 nM Zn^2+^ significantly inhibited responses to 30 μM GABA by ~80% for cells expressing α4β3 subunits, with little effect on GABA currents recorded from α4β3δ- or α4β3γ2L-expressing cells (Supplementary Fig. 2a), suggesting minimal expression of α4β3 receptors when either δ or γ2L is included in the transfection. We confirmed the incorporation of the δ subunit into receptors using the selective potentiator DS2^26^ (5 μM), which potentiated 0.3 μM GABA responses from α4β3δ, but not α4β3-expressing, cells (Supplementary Fig. 2b). Together, these data indicate that when the three subunit cDNAs α4, β3 and δ are co-transfected, the predominant receptor population expressed is α4β3δ, and that this isoform consequently underlies the spontaneous current in these cells.

### Extracellular motifs in β3 subunits are essential for spontaneous activity

**Fig. 2:**
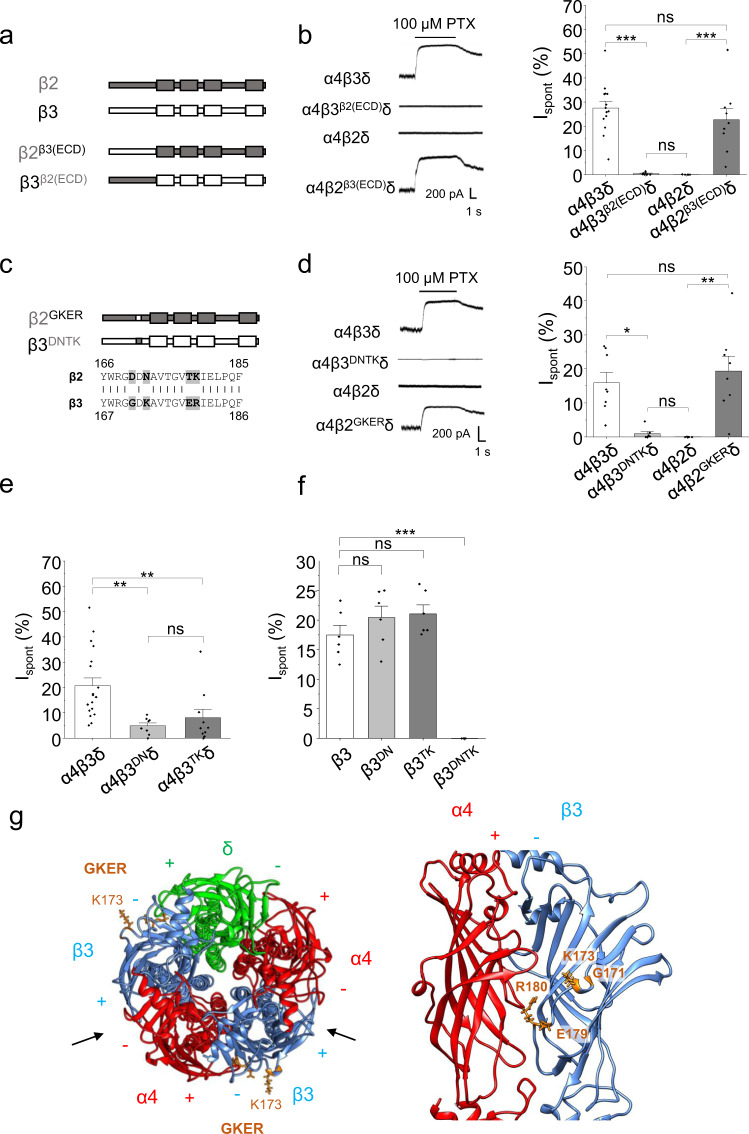
β3 subunit ECD residues responsible for spontaneity of heteromeric GABA_A_Rs. **a** Schematic of β2/3 chimeras with exchanged ECDs. **b** Left: I_PTX_ for wild type and chimeric α4βδ receptors. Right: I_spont_ of wild type and chimeric α4βδ receptors. Wild-type α4β3δ I_spont_ was significantly larger than α4β3^β2(ECD)^δ (***P = 8.7E-7) and no different from α4β2^β3(ECD)^δ (*P* = 0.62). Wild-type α4β2δ was significantly smaller than α4β2^β3(ECD)^δ (***P = 0.00088) and no different from α4β3^β2(ECD)^δ (*P* = 1; one-way ANOVA with Tukey’s post-hoc test; α4β3δ: *n* = 14; α4β3^β2(ECD)^δ: *n* = 8; α4β2δ: *n* = 7; α4β2^β3(ECD)^δ: *n* = 9). **c** Schematic of chimeras with the GKER/DNTK motif exchanged between the β2/3 subunits. Primary sequences for β2 and β3 are shown below with GKER and DNTK residues highlighted. **d** Left: I_PTX_ for wild type and ECD motif-exchanged α4βδ receptors. Right: I_spont_ of wild type and ECD motif-exchanged α4βδ receptors. Wild-type α4β3δ I_spont_ was significantly larger than α4β3^DNTK^δ (*P = 0.038) and no different from α4β2^GKER^δ (*P* = 1). Wild-type α4β2δ was significantly smaller than α4β2^GKER^δ (**P = 0.0010) and no different from α4β3^DNTK^δ (*P* = 1; Kruskall–Wallis with Dunn’s post-hoc test; α4β3δ: *n* = 8; α4β3^DNTK^δ: *n* = 7; α4β2δ: *n* = 7; α4β2^GKER^δ: *n* = 8). **e** I_spont_ of α4β3δ receptors expressing partial exchange of the GKER/DNTK motif. Wild-type α4β3δ I_spont_ was significantly larger than α4β3^DN^δ (**P = 0.0022) and α4β3^TK^δ (**P = 0.0064). Mutant receptors were not significantly different (*P *= 1; Kruskall–Wallis with Dunn’s post-hoc test; α4β3δ: *n* = 19; α4β3^DN^δ: *n* = 8; α4β3^TK^δ: *n* = 10). **f** I_spont_ of wild-type β3 homomeric receptors, and those containing a partial or full exchange of the GKER/DNTK motif. As β3 homomers are insensitive to GABA, pentobarbital (100 μM) was used to generate I_Max_. β3^DNTK^ subunits did not form functional receptors. Wild-type β3 homomers were no different from β3^DN^ (*P* = 0.54) or β3^TK^ (*P* = 0.37; Kruskal–Wallis with Dunn’s post-hoc test; β3: *n* = 6; β3^DN^: *n* = 6; β3^TK^: *n* = 6; β3^DNTK^: *n* = 5). **g** Homology model of the α4β3δ receptor shown in plan view (left) and from the side at the α^+^–β^−^ interface, highlighting the GKER residues in the β3 subunits. Principal (+) and complementary (−) faces are indicated. Arrows show the orthosteric GABA binding sites. Data are presented as mean values ± SEM. *P < 0.05; **P < 0.01; ***P < 0.001; ns no significance. Source data are provided as a Source Data file for Fig. 2.

Key differences in structure between β2 and β3 subunits are likely to underlie why only β3 subunits support spontaneous activity of GABA_A_Rs. To identify the domains involved we created chimeric subunits, by first exchanging the entire N-terminal extracellular domain (ECD) between β2 and β3 subunits (Fig. 2a). This exchange completely switched the spontaneous properties of receptors; replacing β2 ECD with that of β3 in the α4β2^β3(ECD)^δ receptor gave rise to similar levels of spontaneous activity as wild-type α4β3δ, while the converse exchange (α4β3^β2(ECD)^δ) revealed negligible spontaneous currents, similar to wild-type α4β2δ receptors (Fig. 2b; Supplementary Table 1).

To identify key determinants within the ECD that underlie the difference in spontaneous activity between β2- and β3-containing receptors, we focused on one major difference between the β subunits: the ability of β3, but not β2, to form functional homomers. This homomeric assembly is due to four residues in a cassette of ten within the ECD of the β3 subunit: Gly171, Lys173, Glu179 and Arg180, together known as the GKER motif^27^ (specifically, GxKxxxxxER). The equivalent residues in β2 are Asp170, Asn172, Thr178 and Lys179 (DNTK)^27^. Whether the GKER motif is also important for spontaneous activity was assessed using α4β3δ receptors. The β subunit extracellular motifs were exchanged and expressed as α4β3^DNTK^δ and α4β2^GKER^δ (Fig. 2c). For α4β2^GKER^δ receptors, similar levels of spontaneous activity were measured compared to wild-type α4β3δ. By contrast, α4β3^DNTK^δ receptors exhibited negligible spontaneous activity, equivalent to wild-type α4β2δ receptors (Fig. 2d, Supplementary Table 1). Thus, this ECD assembly motif plays a critical role in permitting spontaneous activity in GABA_A_Rs.

To distinguish between its role in mediating homo-oligomerisation and spontaneous gating in heteromeric receptors, β3 subunits were created where each half of GKER was mutated to its β2 equivalent. Dividing DNTK in α4β3^DN^δ and α4β3^TK^δ receptors substantially reduced, but did not ablate, spontaneous activity compared to α4β3δ receptors (Fig. 2e; Supplementary Fig. 1c; Supplementary Table 1). However, when expressed as homomers (β3^DN^ or β3^TK^) there was no disruption to homo-oligomerisation indicated by the unaltered I_spont_ (normalised to 100 μM pentobarbital) compared to wild-type β3 assemblies (Fig. 2f; Supplementary Fig. 1d). Therefore, all four residues are required for full spontaneous activity in α4β3δ heteromers, while either pair of residues is sufficient for β3 homomeric assembly and spontaneous gating. Hence, homo-oligomerisation of β3 subunits and spontaneous gating of heteromeric α4β3δ receptors depend differentially on the GKER motif. Furthermore, these results indicate that the spontaneous current from α4β3δ-transfected cells cannot involve a significant contribution from β3 homomers.

To locate the GKER motif within the α4β3δ GABA_A_R structure, we constructed a homology model (Fig. 2g) derived from the cryo-EM structure of the α1β3γ2 receptor^5^. Studies using both free subunits and concatemeric constructs have suggested that recombinant α4βδ receptors can assemble with various stoichiometries and subunit arrangements^28,29^. However, as both functional and structural data support the inclusion of a single δ subunit in freely assembled recombinant receptors (arranged α-β-α-β-δ clockwise from the extracellular space)^30,31^, this was the most parsimonious basis for our homology model. This revealed the GKER motif positioned at the non-GABA-binding α^+^–β^−^ and δ^+^–β^−^ interfaces, connecting β strands 8 and 9 (Fig. 2g).

### Additional subunits affecting GABA_A_R spontaneous activity

**Fig. 3:**
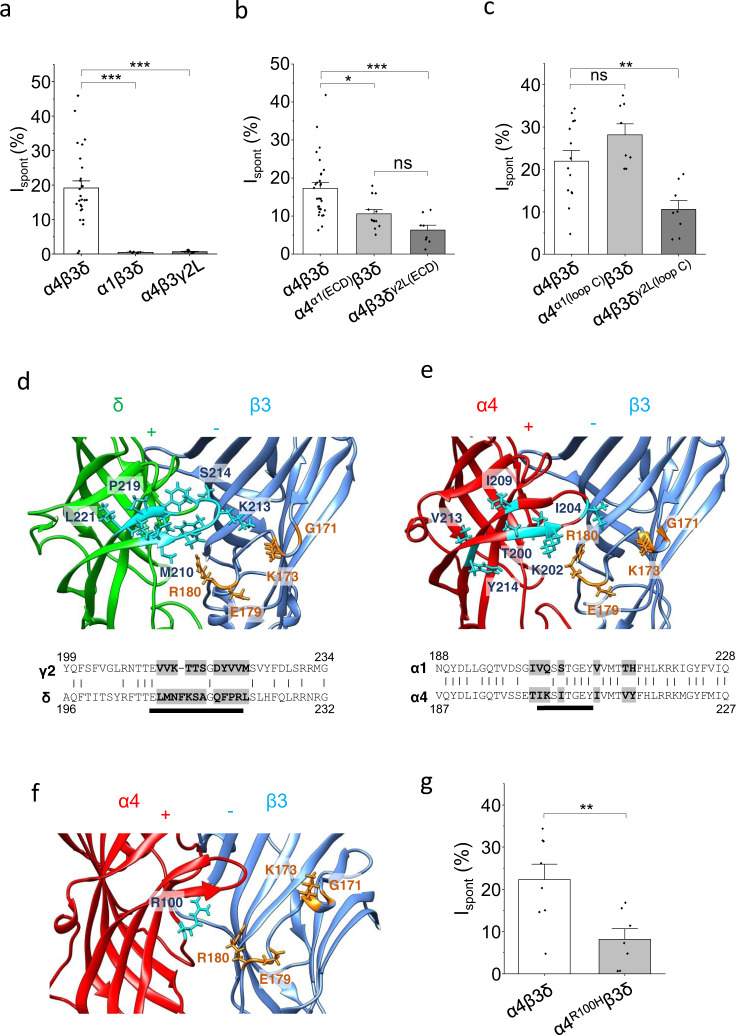
α4 and δ ECDs are important for spontaneous activity. **a** I_spont_ of extrasynaptic-type α4β3δ receptors, and receptors where a single subunit was exchanged for a synaptic-type subunit. I_spont_ was significantly reduced when α1 replaced α4 (***P = 2.4E-5) and when γ2L replaced δ (*P*  =2.5E-6; one-way ANOVA with Tukey’s post-hoc test; α4β3δ: *n* = 27; α1β3δ: *n* = 7; α4β3γ2L: *n* = 10). **b** I_spont_ of α4β3δ receptors and receptors where the α4 or δ ECDs were replaced by those from α1 and γ2L, respectively. I_spont_ was significantly reduced when α4^α1(ECD)^ replaced α4 (*P = 0.014) and when δ^γ2L(ECD)^ replaced δ (***P = 0.00044); there was no difference between the two mutant receptors (*P* = 0.35; one-way ANOVA with Tukey’s post-hoc test; α4β3δ: *n* = 29; α4^α1(ECD)^β3δ: *n* = 12; α4β3δ^γ2L(ECD)^: *n* = 12). **c** Residues that were not conserved in loop C region between α4 and α1, and between δ and γ2L, were exchanged and I_spont_ for α4β3δ receptors incorporating these substitutions (α4^α1(loop C)^ and δ^γ2L(loop C)^) was determined. No effect was observed when introducing loop C residues from α1 into α4 (*P* = 0.21), while a significant reduction was observed when introducing loop C residues from γ2L into δ (**P = 0.0098; one-way ANOVA with Tukey’s post-hoc test; α4β3δ: *n* = 14; α4^α1(loop C)^β3δ: *n* = 8; α4β3δ^γ2L(loop C)^: *n* = 8). Exchanged residues are shown in cyan in the homology models for the δ (**d**) and α4 (**e**) subunit interfacial site opposing the β3 subunit. They are also highlighted in the sequence alignments below. Black bars represent approximate constituent residues of loop C. GKER residues of β3 are shown in orange. **f** α4^+^–β3^−^ subunit interface showing α4 R100. **g** I_spont_ of wild-type α4β3δ receptors was significantly larger than receptors expressing the α4 subunit R100H mutation (**P = 0.0087, two-sided unpaired *t*-test; α4β3δ: *n* = 8; α4^R100H^β3δ: *n* = 7). Data are presented as mean values ± SEM. *P < 0.05; **P < 0.01; ***P < 0.001; ns no significance. Source data are provided as a Source Data file for Fig. 3.

Although β3 subunits are important for spontaneous activity of αβγ and αβδ receptors, it is likely that other subunits also play a role, given that receptors incorporating α4/6 and/or δ subunits showed increased spontaneous activity (Fig. 1b). To investigate, spontaneous currents mediated by α1β3δ and α4β3γ2L receptors were compared to α4β3δ (Fig. 3a; Supplementary Fig. 1a; Supplementary Table 1). Both α1β3δ and α4β3γ2L receptors exhibited negligible spontaneous activity, indicating that incorporation of both α4 (or α6) and δ, together with β3 subunits, is necessary for agonist-independent gating.

To determine if the ECDs of the non-β subunits are important, chimeras were generated which replaced the α4 and δ ECDs with those from α1 (α4^α1(ECD)^) and γ2L (δ^γ2L(ECD)^), respectively. Expressing these chimeric subunits in α4^α1(ECD)^β3δ and α4β3δ^γ2L(ECD)^ receptors reduced, but did not abolish, spontaneous activity (Fig. 3b; Supplementary Table 1). These chimeric receptors did not show the same reduction in spontaneous current as wild-type α1β3δ and α4β3γ2L receptors, suggesting that the ECDs in α4 and δ, while contributing towards spontaneity, are not the only domains in these subunits that control spontaneous gating.

As the β3 GKER motif’s importance for GABA_A_R assembly and spontaneous activity most likely involves interactions with adjacent subunits^27^, we examined residues in the α4 and δ subunit ECDs that could interact with GKER across the α^+^-β^-^ and δ^+^–β^−^ interfaces. We focused on residues surrounding and including loop C of α, δ and γ2L subunits, as this structure projects like a ‘structural clasp’ from the principal subunit side, spanning the subunit interface, and potentially interacting with the complementary face of juxtaposed β subunits. Accordingly, loop C and surrounding residues of the α4 subunit (T200 to L217) were exchanged for the equivalent residues in α1 subunits (I201 to L218). A similar switch of δ subunit residues (T206 to S222) to their equivalents in γ2L (T209 to S224) was also performed. Compared with α4β3δ receptors, spontaneity was unaltered by exchanging the α4 domain with that from α1 (α4^α1(loop C)^β3δ receptors); however, a reduction in spontaneous current was evident after the exchange of the δ domain with that from γ2L (α4β3δ^γ2L(loop C)^ receptors; Fig. 3c; Supplementary Table 1). Notably, the amino acid homology of the exchanged domain is greater between α1 and α4 (61%) compared to γ2L and δ (29%) subunits (Fig. 3d, e).

To search for the structural determinants that could account for why α4 subunits are more effective at inducing spontaneous activity than α1, the α4β3δ receptor model was used to explore sequence variation between these subunits that may underlie differential interaction with the β3 GKER motif. One prominent difference is an arginine residue (R100) in α4 replaced by a histidine (H101) in α1 (Fig. 3f). Expression of α4^R100H^β3δ revealed significantly lower levels of spontaneous activity compared to wild-type α4β3δ (Fig. 3g; Supplementary Table 1), indicating that α4^R100^ alone, and/or its interaction with the β3 GKER motif (possibly through an arg–arg interaction) is important for regulating spontaneous receptor activity. Histidine 101 in α1 is also critical for the binding of benzodiazepines at the α^+^–γ^−^ interface^32^, corroborating this residue’s importance in modulating receptor activity.

In summary, the subunit composition of the pentamer is critical for determining spontaneous activity, with subunit interactions at the α^+^–β^−^ and δ^+^–β^−^ ECD interfaces contributing to the ability of the receptor to gate spontaneously. However, exchanging ECDs did not abolish spontaneous activity, suggesting other determinants exist in α4 and δ subunits.

### Spontaneous gating is associated with increased GABA potency

**Fig. 4:**
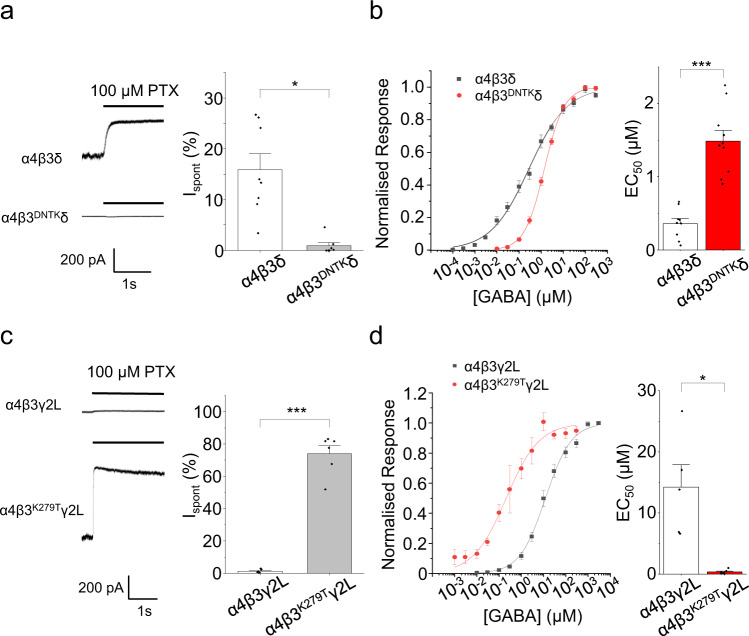
GABA potency is associated with spontaneous gating of GABA_A_Rs. **a** Left: recordings of α4β3δ and non-spontaneous α4β3^DNTK^δ receptors during PTX application. Right: I_spont_ showing significant reduction when GKER is substituted with DNTK. For comparative purposes, data are reproduced here from Fig. 2d. **b** Left: GABA concentration-response curves for α4β3δ and α4β3^DNTK^δ receptors. Right: GABA EC_50_ values (***P = 9E-6, two-sided unpaired *t*-test; corresponding Hill coefficient values are: α4β3δ: 0.5 ± 0.02, *n* = 9; α4β3^DNTK^δ: 1.0 ± 0.04, *n* = 10). **c** Left: recordings of α4β3γ2L and α4β3^K279T^γ2L receptors during PTX application. Right: I_spont_ revealing that K279T in the M2–M3 linker significantly increases spontaneous activity (***P = 2.5E-5, two-sided unpaired *t*-test; *n* = 6). **d** Left: GABA concentration-response curves for wild-type α4β3γ2L and α4β3^K279T^γ2L receptors. Right: GABA EC_50_ values (*P = 0.020, two-sided unpaired *t*-test; corresponding Hill coefficient values are: α4β3γ2L: 0.8 ± 0.03, *n* = 5; α4β3^K279T^γ2L: 0.8 ± 0.2, *n* = 6). Data are presented as mean values ± SEM. *P < 0.05; ***P < 0.001. Source data are provided as a Source Data file for Fig. 4.

To identify the mechanisms underlying spontaneity, the relationship between ligand-gating and spontaneous activity was investigated. If receptors are capable of opening in the absence of agonist, then it is plausible they may be more responsive to agonist^33^. GABA concentration-response curves were constructed for wild-type α4β3δ/γ2L receptors, and for corresponding mutant receptors with altered spontaneous activity. The first receptor variant selected was the non-spontaneous α4β3^DNTK^δ (Fig. 4a). The GABA concentration-response curve for α4β3^DNTK^δ was right-shifted compared with wild-type α4β3δ receptors (EC_50_s of 1.5 and 0.4 μM, respectively; Fig. 4b). To explore further, the mutation K279T in the β3 M2–M3 linker was used since this markedly increases GABA potency at α1β3γ2L receptors^34^. For α4β3^K279T^γ2L, high levels of spontaneity were apparent compared to negligible constitutive activity for α4β3γ2L (Fig. 4c, Supplementary Table 1). GABA was also significantly more potent at α4β3^K279T^γ2L, with an EC_50_ 35-fold lower (0.4 μM) compared to wild type (14 µM; Fig. 4d).

These experiments suggest that spontaneous activity is associated with increased GABA potency, potentially reflecting lowered activation energy for channel opening. This is consistent with δ-containing receptors, which are more sensitive to GABA, also displaying greater spontaneous activity than their γ-containing counterparts (Fig. 1d).

### Phosphorylation status of β3 subunits affects spontaneous activity

**Fig. 5:**
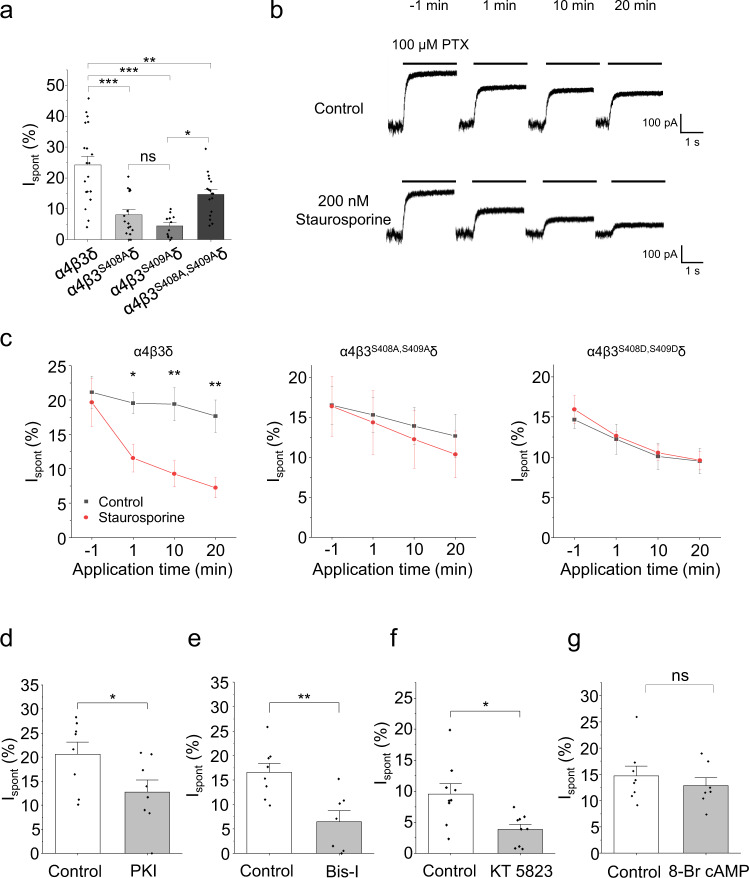
Phosphorylation of β3 subunits regulates spontaneous activity. **a** I_spont_ of receptors incorporating β3 subunits where either or both phosphorylation sites, S408 and S409, have been mutated. Wild-type α4β3δ was significantly more spontaneous than α4β3^S408A^δ (***P = 3.5E-6), α4β3^S409A^δ (***P = 2.1E-7) and α4β3^S408A,S409A^δ (**P = 0.0074). Individually mutated receptors were no different from each other (*P* = 0.68), and the double mutant was statistically more spontaneous than α4β3^S409A^δ (*P = 0.012) but not α4β3^S408A^δ (*P* = 0.13, one-way ANOVA with Tukey’s post-hoc test; α4β3δ: *n* = 19; α4β3^S408A^δ: *n* = 16; α4β3^S409A^δ: *n* = 12; α4β3^S408A,S409A^δ: *n* = 17). **b** Recordings of α4β3δ receptors exposed to PTX at several time points before and during application of either control (DMSO-containing) solution or the protein kinase inhibitor staurosporine (200 nM). **c** I_spont_ during a 20 min application of either DMSO control or 200 nM staurosporine for wild-type, phospho-null α4β3^S408A,S409A^δ and phosphomimetic α4β3^S408D,S409D^δ receptors. Only wild-type α4β3δ receptors displayed smaller I_spont_ during staurosporine application after 1, 10 and 20 min (*P = 0.019; **P = 0.0036, **P = 0.0029, mixed ANOVA with Tukey’s post-hoc test; *n* = 6; α4β3^S408D,S409D^δ + staurosporine: *n* = 5). **d** Including selective kinase inhibitors in the patch electrode solution significantly reduced spontaneous currents of α4β3δ receptors: PKA inhibitor peptide 14–22 (PKI, 1 μM; *P = 0.043; *n* = 8), **e** bisindolylmaleimide-I (Bis-I, 200 nM; **P = 0.0039; control: *n* = 8; Bis-I: *n* = 7) and **f** KT 5823 (1 μM; **P = 0.0076; *n* = 9) to inhibit PKA, PKC and PKG, respectively. Internally applied PKA-activator 8-Br cAMP (1 mM) did not affect spontaneous activity (*P* = 0.46; control: *n* = 8; 8-Br cAMP: *n* = 7). Kinase modulators were compared with appropriate vehicle controls. All comparisons from **d**–**g** were two-sided unpaired *t*-tests, Data are presented as mean values ± SEM. *P < 0.05; **P < 0.01; ***P < 0.001; ns no significance. Source data are provided as a Source Data file for Fig. 5.

The large intracellular domain (ICD) between M3 and M4 of GABA_A_Rs is a location for phosphorylation by protein kinases and for modifying receptor trafficking and function^35–38^, which could be important for controlling spontaneous activity. Indeed, activation of protein kinases can increase spontaneous currents mediated by α4β3δ receptors^18^, but the phosphorylation site is unknown. We investigated phosphorylation of the β3 subunit, given its importance for spontaneous activity and that phosphorylation sites have been identified involving adjacent serines, S408 and S409, within the large ICD^36^. Using serine to alanine substitutions to prevent β3 phosphorylation in α4β3^S408A^δ and α4β3^S409A^δ receptors, spontaneous activity was reduced by 67% and 82%, respectively. Mutating both residues (α4β3^S408A,S409A^δ) reduced the spontaneous current by 39% (Fig. 5a, Supplementary Fig. 3a; Supplementary Table 1). To ensure these effects were due to receptor phosphorylation status, the cell-permeable, broad-spectrum kinase inhibitor staurosporine (200 nM) was applied to α4β3δ receptors and the I_spont_ measured over 20 min. Consistent with previous studies showing rapid effects of kinase inhibitors on spontaneous current^18^, I_spont_ decreased significantly after 1 min exposure and declined further during the 20 min window. Despite some rundown in spontaneous activity under control conditions, I_spont_ was significantly smaller in staurosporine compared to control (Fig. 5b, c). To confirm that staurosporine was affecting the phosphorylation status of S408 and S409, it was also applied to phospho-null α4β3^S408A,S409A^δ, and phosphomimetic α4β3^S408D,S409D^δ receptors (Fig. 5c) where it had no effect, with I_spont_ similar to control.

Both S408 and S409 are phosphorylated by various serine/threonine kinases, including cAMP-dependent protein kinase (PKA), protein kinase C (PKC), protein kinase G (PKG) and Ca^2+^/calmodulin-dependent protein kinase II (CaMKII)^36,38–40^. To identify whether specific protein kinases are responsible for regulating the spontaneous current, the following selective kinase inhibitors were used: PKA inhibitor peptide 14–22 (1 µM), bisindolylmaleimide-I (200 nM) and KT 5823 (1 µM) to inhibit PKA, PKC and PKG, respectively. Inhibitors were individually included in the patch electrode solution and allowed to dialyse into the cell for 2 min before I_spont_ was measured. Each inhibitor reduced the spontaneous current of α4β3δ receptors compared to controls (Fig. 5d–f; Supplementary Fig. 3b). However, internal application of the PKA-activator 8-Br cAMP (1 mM) did not affect I_spont_ (Fig. 5g). This accords with the lack of difference in I_spont_ between α4β3^S408D,S409D^δ and wild-type α4β3δ receptors (Supplementary Fig. 3c), suggesting that S408 and S409 are fully phosphorylated under control conditions.

Overall, these results demonstrate that PKA, PKC and PKG can target serine phosphorylation sites to modulate α4β3δ receptor-mediated spontaneous currents. Phosphorylation by CaMKII was also examined since this kinase can phosphorylate not only S408 and S409 but also S383^39,40^. However, the β3^S383A^ mutant had no effect on spontaneous activity in α4β3^S383A^δ receptors compared with wild type (Supplementary Fig. 3d, Supplementary Table 1). Moreover, internally applying the selective CaMKII inhibitor KN-62 (3 µM) also had no effect on I_spont_ (Supplementary Fig. 3b, e). These results imply that CaMKII activity is unlikely to modulate spontaneous activity of recombinant receptors expressed in HEK cells, although this may differ in a neuronal environment^41^.

### Allosteric modulators potentiate GABA_A_R currents in the absence of an orthosteric agonist

Neurosteroids are widely recognised as important endogenous allosteric modulators of GABA_A_Rs, regulating both extrasynaptic and synaptic inhibition^42^. We therefore assessed the effect of the naturally occurring positive modulatory neurosteroids tetrahydro-deoxycorticosterone (THDOC) and allopregnanolone on wild-type and mutant extrasynaptic receptors that exhibit distinct levels of spontaneous activity. Neurosteroids were applied until the holding current reached steady state and, after wash-out, saturating concentrations of GABA and PTX were applied. The change in the holding current in response to neurosteroids was added to I_PTX_ for the subsequent calculation of I_spont_ (see Methods).

**Fig. 6:**
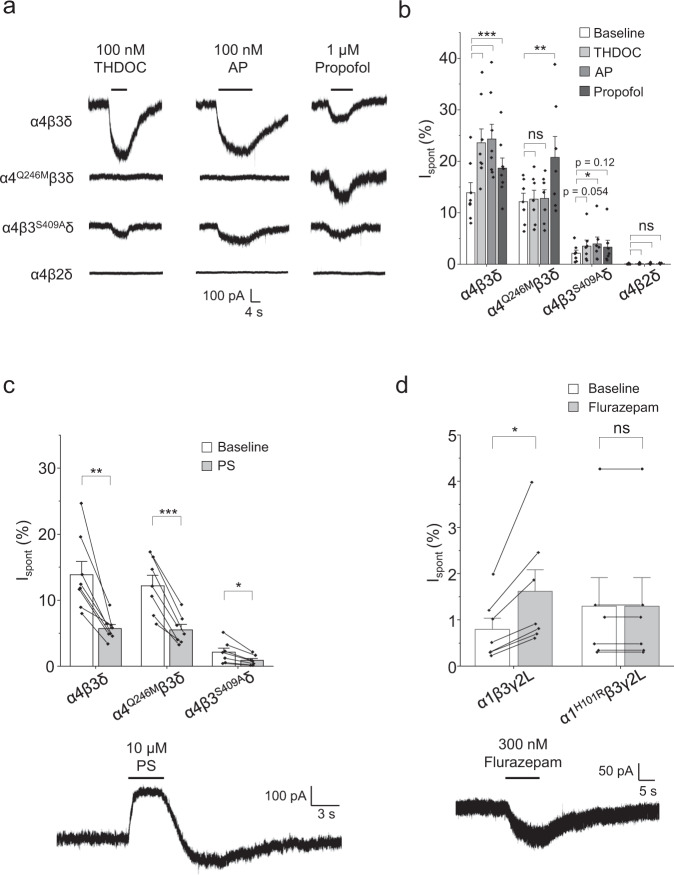
Allosteric modulators of GABA_A_Rs regulate spontaneous currents. **a** Recordings of various receptor isoforms exposed to the positive allosteric modulators THDOC (100 nM), allopregnanolone (AP, 100 nM) and propofol (1 μM). **b** Modulator-induced changes in holding current I_Mod_ were measured, before saturating GABA (500 μM) and PTX (100 μM) were applied. The I_Mod_ was added to the I_PTX_ to calculate I_spont_ in the presence of the modulators (see Methods). Wild-type α4β3δ I_spont_ was potentiated by THDOC (***P = 4.7E-8), AP (***P < 1.0E-9) and propofol (***P = 0.00029; *n* = 8). The neurosteroid-insensitive α4^Q246M^β3δ was not affected by THDOC (*P* = 1.0) or AP (*P* = 0.99), but was potentiated by propofol (**P = 0.0028; *n* = 7). The little-spontaneous α4β3^S409A^δ was potentiated by all modulators, but the effect was not statistically significant for some (THDOC: *P* = 0.054, AP: *P = 0.011, propofol: *P* = 0.12; *n* = 7). The non-spontaneous α4β2δ was not potentiated by any modulator (*P* ≥ 0.63; *n* = 5). All comparisons used one-way repeated measures ANOVA with Tukey’s post-hoc test. **c** Pregnenolone sulfate (PS, 10 µM) reduced spontaneous currents of α4β3δ (**P = 0.0023; *n* = 8), α4^Q246M^β3δ (***P = 0.00060; *n* = 7) and α4β3^S409A^δ receptors (*P = 0.011; *n* = 7; two-sided paired *t*-tests). Representative holding current of α4β3δ during PS application is shown below. **d** Flurazepam (300 nM) potentiated spontaneous currents of α1β3γ2L receptors (*P = 0.011; two-sided paired *t*-test; *n* = 7), but not for the benzodiazepine-insensitive α1^H101R^β3γ2L receptor (P = 1.0; two-sided Wilcoxon signed-rank test; *n* = 6). Representative current of wild-type α1β3γ2L during flurazepam application is shown below. Data are presented as mean values ± SEM. *P < 0.05; **P < 0.01; ***P < 0.001; ns no significance. Source data are provided as a Source Data file for Fig. 6.

Applying either neurosteroid, at 100 nM, increased the α4β3δ holding current by over 70% (Fig. 6a, b) in the absence of an orthosteric ligand. Effectively, these agents were acting as allosteric agonists of the receptor. Although neurosteroids will directly activate GABA_A_Rs at high concentrations, at 100 nM there was no activation of the non-spontaneous α4β2δ receptor (Fig. 6a, b), indicating this effect of the neurosteroids is restricted to receptors displaying a propensity to open spontaneously. To establish whether the neurosteroids were acting via their binding site on GABA_A_Rs, we utilised the neurosteroid-insensitive α4 mutant, Q246M, in α4^Q246M^β3δ receptors^43,44^. While this mutation had no effect on basal spontaneous current, it ablated the neurosteroid allosteric agonist activity (Fig. 6a, b). We also examined neurosteroid actions at α4β3^S409A^δ receptors since this phosphorylation mutant displays significantly impaired spontaneous activity. Although basal spontaneous currents were much smaller compared to wild type, THDOC and allopregnanolone acted as weak allosteric agonists (Fig. 6a, b), demonstrating that the effects of the positive modulatory neurosteroids correlate with the level of spontaneous activity.

Sulphated neurosteroids, such as pregnenolone sulphate (PS), are also synthesised in the brain but, unlike their potentiating counterparts, they inhibit GABA-induced current^45^. PS (10 µM) is a negative allosteric modulator and inhibited spontaneous holding currents of α4β3δ, α4^Q246M^β3δ and α4β3^S409A^δ receptors (Fig. 6c). This inhibition was unaffected by the α4 Q246M mutation due to PS having a binding site distinct from the positive modulatory neurosteroids^44^.

GABA_A_Rs are also modulated by general anaesthetics. The most widely used intravenous anaesthetic propofol is a potent positive allosteric modulator acting via a discrete binding site to those for potentiating and inhibitory neurosteroids^46^. Recording from α4β3δ, α4^Q246M^β3δ and α4β3^S409A^δ receptors revealed that propofol (1 µM), as with neurosteroids, displayed allosteric agonism, increasing the holding current of all these receptor isoforms but significantly with no effect on the non-spontaneous α4β2δ receptor (Fig. 6a, b).

Benzodiazepines are subtype-selective modulators of GABA_A_Rs, potentiating α1-3,5βxγ2 isoforms via their main binding site located at the α^+^–γ^−^ subunit interface^47,48^. Despite the low level of spontaneity of α1β3γ2L receptors, the holding current was increased by flurazepam (300 nM), but not in cells expressing the benzodiazepine-insensitive α1^H101R^β3γ2L receptor (Fig. 6d)^32^. This concurs with previous work reporting that benzodiazepines can enhance spontaneous currents of GABA_A_Rs^11,12^.

**Fig. 7:**
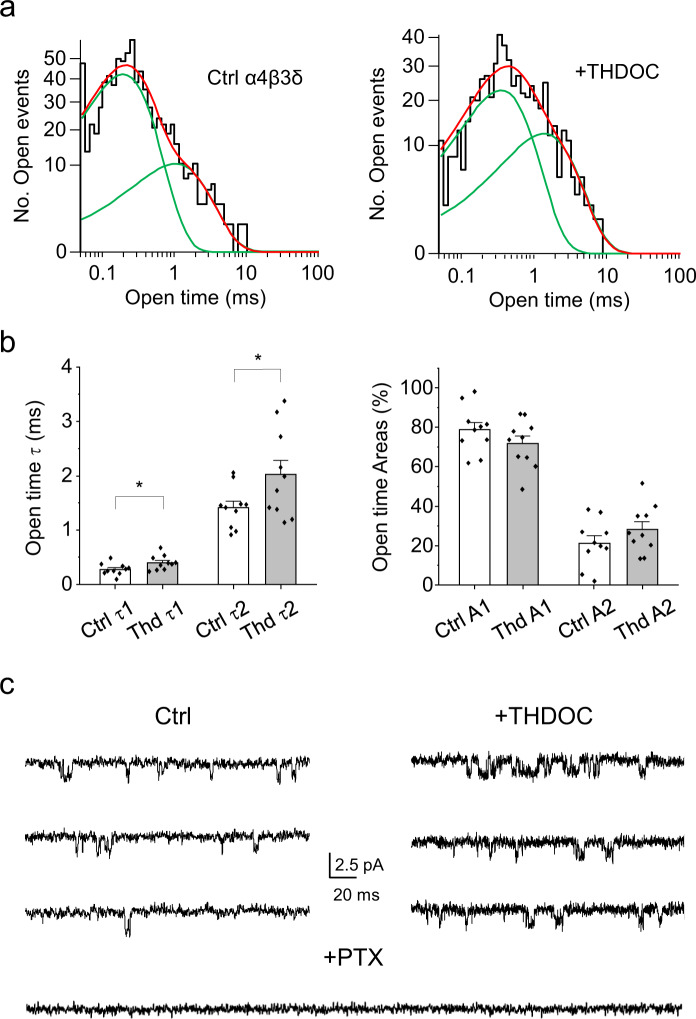
THDOC prolongs the open states for spontaneous α4β3δ currents. **a** Open state distributions compiled for spontaneous single-channel currents recorded from α4β3δ receptors expressed in HEK cells in the absence of GABA, as a control, and in the presence of 100 nM THDOC. Green lines show individual exponential component fits, while red lines depict the overall summed fit from the mixture of exponentials. **b** Bar graphs for the exponential open time constants τ1 and τ2 (left panel) and their respective areas, A1 and A2 (right panel). THDOC increased τ1 (*P = 0.041) and τ2 (*P = 0.048) but did not significantly affect the areas (*P* = 0.30; two-sided paired *t*-tests; *n* = 10). **c** Example epochs of spontaneous single-channel recording for α4β3δ receptors obtained from the same outside-out patch under control conditions (zero GABA, left panel), and in the presence of 100 nM THDOC (right panel) or 100 μM PTX (lower trace). Calibration bars apply to all traces. Data are presented as mean values ± SEM. *P < 0.05. Source data are provided as a Source Data file for Fig. 7.

Our results demonstrate that physiologically relevant concentrations of both positive and negative allosteric modulators can affect GABA_A_R-mediated currents in the absence of orthosteric agonism. To explore the underlying mechanism, we used single-channel recording from α4β3δ receptors expressed in outside-out patches from HEK cells. Recording in the absence of GABA revealed spontaneous single-channel currents (Fig. 7c) that were modulated by 100 nM THDOC and blocked by 100 μM PTX. Spontaneous currents exhibited brief open durations, rarely forming bursts of channel activity. Such bursts that were evident usually occurred in the presence of THDOC (Fig. 7c). The open time distributions were best described by the sum of two exponential components and the respective time constants (τ1, τ2) were significantly increased by 100 nM THDOC (Fig. 7a–c). Our recordings suggest that more than one channel was often present in each patch (occasional channel stacking), so no closed time analysis was attempted, especially given the paucity of bursts (Fig. 7c). The proportion of channel openings in the short and long open time distributions (A1 and A2, respectively) suggested that 100 nM THDOC may promote increased openings in A2 at the expense of those in A1, although this was noted only as a trend (Fig. 7b).

In summary, both our macroscopic and single-channel currents reveal that allosteric modulation of GABA_A_R activity occurs in the absence of an orthosteric agonist. As for the potentiation of GABA-gated receptors, the positive modulators we describe here increase the open probability of the GABA channel, shifting the equilibrium of the receptor population, resulting in a greater proportion of receptors in the open conformation at any given time. Consistent with a recent study showing that the benzodiazepine flurazepam enhances spontaneous GABA_A_R currents by prolonging single-channel openings^19^, our single-channel recordings indicate that potentiating neurosteroids also prolong spontaneous open channel durations. Therefore, these modulators are likely to impact on tonic inhibition mediated by these activated receptors in vivo, even in the absence of GABA.

### Spontaneous GABA_A_R activity in hippocampal neurons is regulated by β3 subunits

**Fig. 8:**
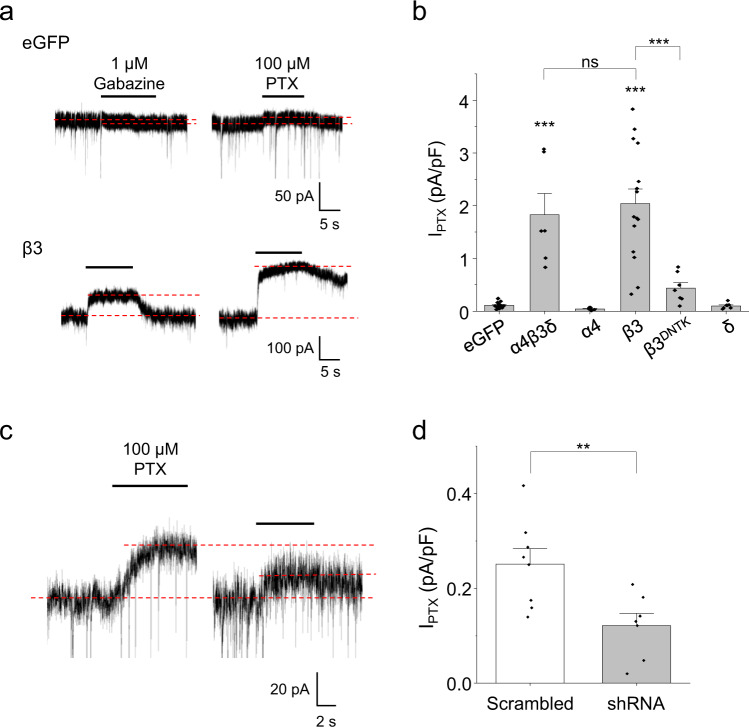
β3 subunit controls spontaneous currents in hippocampal neurons. **a** Recordings from neurons transfected with control eGFP or β3 cDNA and exposed to gabazine (1 μM) and PTX (100 μM). Dashed lines indicate the holding current before and during drug application. Gabazine exhibited a small agonist effect with eGFP-transfected neurons, and partial antagonism at β3-transfected neurons. Both gabazine and PTX were applied until the holding current reached a steady state (~3–5 s). **b** Neurons transfected with eGFP alone or with α4 or δ cDNA showed small PTX-sensitive spontaneous current densities. Transfection with either α4, β3 and δ DNAs together (***P = 1.3E-7) or β3 alone (***P = 4.9E-5) increased spontaneous current densities. Neurons transfected with non-spontaneous β3^DNTK^ cDNA had spontaneous currents that were not significantly greater than eGFP controls (*P* = 0.73), and substantially less than wild-type β3 (*P = 0.014; one-way ANOVA with Tukey’s post-hoc test; eGFP: *n* = 16; α4β3δ: *n* = 5; α4: *n* = 5; β3: *n* = 6; β3^DNTK^: *n* = 7; δ: *n* = 6). **c** Hippocampal neurons were transfected with two shRNAs selective for rat β3 knockdown, or with a scrambled control. Recordings of neurons transfected with scrambled control (left) or shRNAs (right) during PTX application are shown. **d** PTX-sensitive spontaneous current densities of neurons transfected with the scrambled control were significantly higher than those transfected with shRNAs to knockdown β3 subunit expression levels (**P = 0.0092; two-sided unpaired *t*-tests; scrambled: *n* = 8; shRNA: *n* = 7). Data are presented as mean values ± SEM. **P < 0.01; ***P < 0.001; ns no significance. Source data are provided as a Source Data file for Fig. 8.

To investigate whether β3 subunits affect spontaneous GABA_A_R activity in a physiological context, α4, β3 and δ subunits were expressed individually or combined in cultured hippocampal neurons. Only neurons transfected with cDNAs for α4β3δ or β3 displayed larger spontaneous currents (determined by applying 100 μM PTX to block all GABA_A_R-mediated current and 1 μM gabazine to block GABA-mediated current) compared to eGFP-expressing control neurons (Fig. 8a, b; Supplementary Fig. 4a). Spontaneous currents for α4β3δ and β3 cDNA-transfected neurons were similar in amplitude, suggesting that the level of β3 expression is the predominant determinant for the formation of spontaneously active neuronal GABA_A_Rs. The requirement for the β3 GKER motif was also evident following expression of the β3^DNTK^ variant which markedly reduced spontaneous activity below that for wild-type receptors (Fig. 8b; Supplementary Fig. 4a). The β3 subunit and GKER motif are therefore important in promoting spontaneous receptor activity in neurons, consistent with our recombinant receptor data.

Spontaneous currents in neurons transfected with only β3 cDNA were partly blocked by gabazine (1 μM), indicating the formation of heteromeric, rather than homomeric, receptors (Fig. 8a). Preferential expression of heteromers was also supported by larger whole-cell currents in response to GABA (1 μM) recorded from β3-transfected neurons compared to eGFP controls (Supplementary Fig. 4b). Crucially, the ratio of gabazine to PTX-induced currents was not significantly different between β3 and α4β3δ-expressing neurons (Supplementary Fig. 4c) indicating that these neurons most likely express similar relative populations of these receptors and that β3 subunit expression is a limiting factor. Importantly, the gabazine/PTX ratio was also comparable with that obtained for the block of spontaneous currents recorded from α4β3δ-expressing HEK cells (Supplementary Fig. 4c), strongly suggesting that the PTX-sensitive currents in neurons must be GABA-independent. We confirmed that spontaneous activity of eGFP- and β3-expressing neurons was independent of ambient GABA derived from synaptic release by incubation with the vacuolar H^+^-ATPase inhibitor concanamycin A (0.5 μM), which reduces vesicular GABA uptake and abolished IPSCs without affecting PTX-sensitive spontaneous currents (Supplementary Fig. 4d, e).

To further explore the importance of β3 subunits in promoting spontaneous activity in neurons, selective short hairpin RNAs (shRNAs) were used to reduce β3 subunit expression. Knockdown of native β3 subunits was confirmed with confocal immunofluorescent imaging (Supplementary Fig. 4f) and was accompanied by over 50% reduction in spontaneous current, compared with neurons transfected with a scrambled shRNA control (Fig. 8c, d). The residual spontaneous currents are likely to reflect incomplete knockdown of the β3 subunit.

**Fig. 9:**
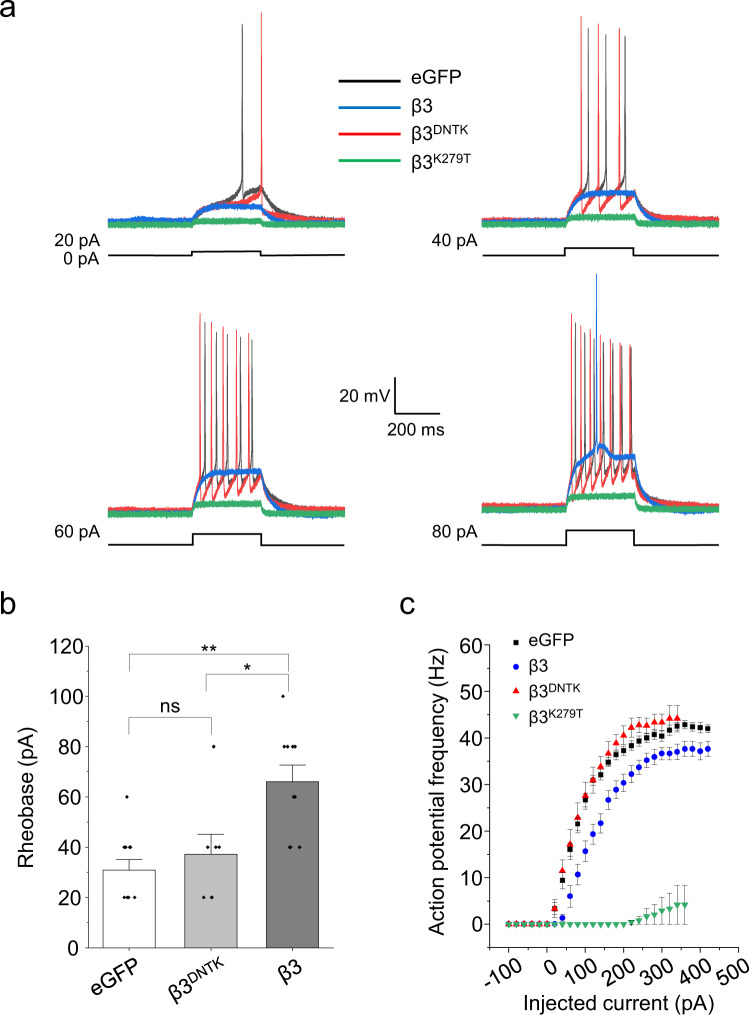
Spontaneously active β3-containing receptors reduce neuronal excitability. **a** Current clamp recordings from hippocampal neurons expressing eGFP, β3, β3^DNTK^ or β3^K279T^ during injection of depolarising constant current steps of increasing amplitude (20 pA increments). **b** Rheobase determination for hippocampal neurons transfected with eGFP, β3 or β3^DNTK^ cDNAs. Neurons expressing β3^DNTK^ showed rheobase values similar to eGFP control (P = 1), while β3-transfected neurons were less excitable, as demonstrated by higher rheobase values than eGFP (**P = 0.0026) and β3^DNTK^-transfected (*P = 0.042) neurons. Expression of the highly spontaneous β3^K279T^ resulted in 7 of 8 neurons unable to evoke action potentials. The one excitable cell had a rheobase of 220 pA (not shown). The input–output curves are shown in **c**. Kruskal–Wallis with Dunn’s post-hoc test were used to compared rheobase values (eGFP: *n* = 11; β3^DNTK^: *n* = 7; β3: *n* = 10; β3^K279T^: *n* = 8). Data are presented as mean values ± SEM. *P < 0.05; **P < 0.01; ns no significance. Source data are provided as a Source Data file for Fig. 9.

The impact of β3-driven spontaneous currents on neuronal excitability was studied using current clamp recording of action potentials evoked by depolarising current steps. Neurons were transfected with cDNAs for either β3, the non-spontaneous β3^DNTK^ or the highly spontaneous β3^K279T^ subunit (Fig. 9). There was neither a shift in the input–output (I–O) relationship for spike firing nor a change in the spike threshold (rheobase) for β3^DNTK^ neurons compared to eGFP controls. However, for β3-expressing neurons, the I–O curve was displaced to a higher rheobase. By contrast, β3^K279T^-expressing neurons were largely silent, with only 1/8 cells firing in response to a large depolarising current (>220 pA). These results indicate that β3 subunits supporting spontaneously active receptors can substantially reduce the level of neuronal excitability.

### Spontaneous activity in different brain regions

**Fig. 10:**
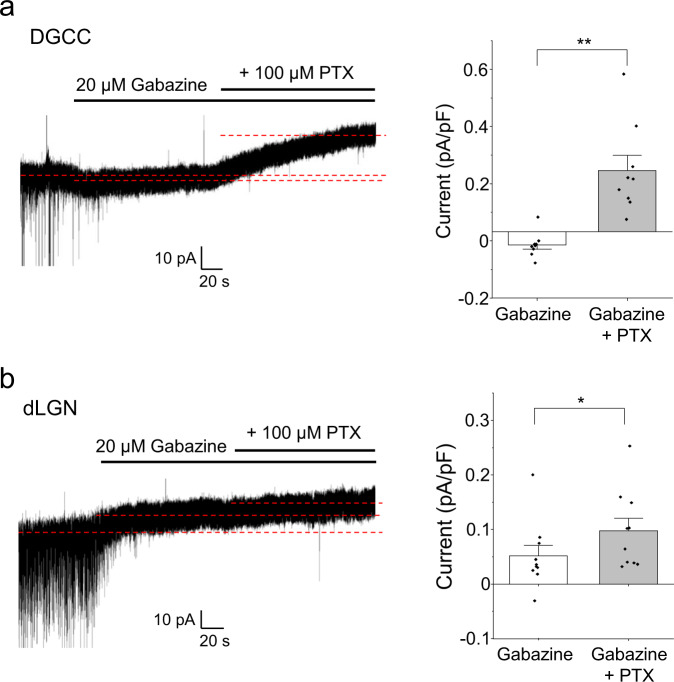
Tonic inhibition in hippocampal and thalamic neurons is differentially dependent on spontaneous gating of GABA_A_Rs. **a** Left: current recording from a dentate gyrus granule cell (DGGC) in an acute hippocampal brain slice showing changes to baseline current when gabazine (20 µM) and PTX (100 µM) were applied. Right: Holding current density changes during antagonist application (**P = 0.0018; two-sided paired *t*-test; *n* = 9). **b** Left: current recording from a relay neuron of the dorsal lateral geniculate nucleus (dLGN) in an acute thalamic brain slice. Right: current density changes during antagonist application (*P = 0.032; two-sided paired *t*-test; *n* = 10). Data are presented as mean values ± SEM. *P < 0.05; **P < 0.01. Source data are provided as a Source Data file for Fig. 10.

After exploring the properties of spontaneously active receptors in heterologous expression systems and cultured neurons^12,18^, it was important to establish whether a similar phenomenon existed in acute brain slices with intact circuitry^13^. We recorded from two regions: rat dentate gyrus granule cells (DGGCs) and dorsal lateral geniculate nucleus (dLGN) thalamic relay neurons, since both express δ subunit-containing GABA_A_Rs^49–51^. To pharmacologically dissect the tonic current in these neurons, gabazine (20 µM) was used to block the GABA-mediated component of the tonic current, and the residual current, blocked by PTX (100 μM), was designated as the spontaneous component. Drugs were applied until steady-state currents were attained. In DGGCs, small increments in holding current were caused by gabazine, consistent with an agonist effect previously reported^13^, followed by a large reduction in holding current with PTX (Fig. 10a). In contrast, tonic currents of thalamic relay neurons were part-inhibited by gabazine, leaving a residual current blocked by PTX (Fig. 10b). We conclude that tonic current in DGGCs is primarily mediated by spontaneous GABA_A_R activity, consistent with an earlier study^13^, whereas in dLGN thalamic relay neurons, tonic current is more reliant on GABA-mediated activity.

## Discussion

In this study we have examined a relatively unexplored area of tonic inhibition involving the contribution of spontaneously active GABA_A_Rs. Although this feature of GABA_A_Rs has been noted, we have little insight into its properties and regulation^12–14,17,18^, including which receptor isoforms are spontaneously active and whether the phosphorylation state of the receptor is crucial^18,52,53^. Here, we demonstrate how spontaneity is reliant upon receptor subunit composition, post-translational modification, and also the level of allosteric modulation. Furthermore, under physiological conditions, we demonstrate that receptor spontaneity is likely to impact upon neuronal excitability via its contribution towards tonic current.

The β subunit is a key assembly component of GABA_A_Rs^27,54^. Despite the considerable homology between β subunits, only β3 was identified as being essential for spontaneity. Nevertheless, other subunits are also important, exemplified by α4β3δ receptors displaying higher levels of spontaneity than α1β3γ2L. Furthermore, single subunit exchanges, such as α1 for α4, or γ2L for δ, are sufficient to reduce spontaneous activity. Our molecular studies identified the GKER motif in the β3 ECD as a critical factor for the expression of spontaneity. Within a pentamer, this motif is located to the complementary (−) side of the subunit interface and, given its role in β3 homo-oligomerisation, is likely to interact with residues on the adjacent subunit’s principal face^27^. Structural models predict that GABA binding ‘locks’ the β^+^–α^−^ interface into a compact conformation, pulling the subunits closer together to widen the channel pore^48^. Given that the GKER motif is likely to contact residues on adjacent subunits^27^, its presence may strengthen these interactions, facilitating channel opening in the absence of bound agonist. This may also explain the requirement for specific receptor subunit compositions (α4/6 and δ) for spontaneity, as GKER interactions at two interfaces may be necessary to lower the activation energy sufficiently to promote spontaneous channel opening. Indeed, expressing β3 subunits with half-substituted GKER motifs, β3^DN^ and β3^TK^, was sufficient to significantly reduce spontaneous currents in heteromeric receptors, but had no effect on the spontaneous currents of homomeric receptors. Presumably, a half-substituted motif may be capable of promoting spontaneity when there are five β^+^–β^−^ interfaces, but not when only two (α^+^–β^−^ and δ^+^–β^−^) exist in heteromers. The importance of non-orthosteric subunit interfaces for modulating receptor activity is highlighted by the activity of benzodiazepines and by novel compounds that target α^+^-β^−^ interfaces^44,46,55,56^.

The GKER motif lies within binding loop F of the β subunit^5^. The role of loop F in Cys-loop receptors is still to be fully defined but may involve transducing ligand-binding to channel gating^57–59^. Interestingly, residues within this loop of the β2 subunit stabilise the closed state of α1β2γ2 receptors, and mutations can modify GABA potency and induce spontaneous gating^60^. The impact of GKER on spontaneous gating and agonist sensitivity, combined with its distance from the GABA binding site, suggests a role in signal transduction. A link between constitutive gating and agonist responsiveness has been previously observed^61–63^, with mutation of the 9’ leucine in the pore-lining M2 region of β3 subunits increasing spontaneous activity and agonist potency^31^. The enhanced sensitivity to agonists, linked to constitutive activity, may promote GABA-mediated transmission via extrasynaptic and perisynaptic GABA_A_Rs, which are exposed to only low concentrations of GABA.

Spontaneous tonic currents displayed considerable variability in cultured and ex vivo neurons. Different expression levels of the β3 subunit will have a profound impact on spontaneous GABA_A_R activity and neuronal excitability evidenced by the degree of spontaneity in hippocampal DGGCs, which express high levels of β3^49^. Furthermore, our recombinant receptor study indicates that extrasynaptic-type α4/δ-containing receptors generate the largest spontaneous currents, and these receptors are also responsible for the majority of tonic inhibition in DGGCs^51^. Indeed, with physiological ambient GABA levels, almost all the tonic current in the DG is produced by spontaneous receptor activation^13^. The reduced spontaneous activity apparent in thalamic relay neurons is also consistent with reduced β3 compared to β1/2 expression and assembly in their extrasynaptic α4βδ receptors^49,50^. This also accords with prior studies suggesting presynaptic GABA release underpins the tonic current in thalamic relay neurons^64^.

Although our use of PTX, a non-subunit-selective inhibitor of GABA_A_Rs, does not unequivocally identify β3-containing receptors as being responsible for spontaneous activity, it is highly probable, given our recombinant receptor data and shRNA knockdown studies, that β3 subunits dictate spontaneous activity in vivo. We would predict that other areas of the brain exhibiting high expression of β3, alongside strong α4/6 and δ subunit expression, should also present tonic currents dependent upon spontaneous receptor activity, e.g. striatal medium spiny neurons possess δ-GABA_A_R-dependent tonic currents^65^ and robustly express β3 over β1/2 subunits^49,66^. Indeed, the importance of β3 subunits for tonic inhibition in these neurons was affirmed using conditional β3 knockout studies, in which tonic inhibition was severely impaired and neuronal excitability enhanced^66^.

Complementing the role of subunit composition, we also demonstrated that spontaneous activity is subject to receptor modulation by protein kinases, neurosteroids, benzodiazepines and general anaesthetics. Analysing spontaneous single-channel currents indicated that THDOC prolonged the existing open channel states (τ1, τ2) and did not cause the appearance of new additional exponential components in the dwell-time distribution^19^. This suggests that neurosteroids are probably not inducing new kinetically distinct channel states per se, but instead most likely modulating already activated (spontaneous) channels. The dependence of spontaneity on receptor phosphorylation state was evident by mutating subunit phosphorylation sites, and by its modulation with protein kinases. This accords with previous work showing PKA activation enhances spontaneous currents mediated by α4β3δ receptors by increasing single-channel opening frequency^18^. In the present study, spontaneous activity depended upon phosphorylation of β3 S408 and S409 and only receptors phosphorylated at both serines displayed maximum spontaneous gating. Intriguingly, phosphorylation of either S408 or S409 enhances the potentiation by neurosteroids and, reciprocally, neurosteroids promote β3 subunit phosphorylation^67^. Phosphorylation of β subunits has multiple effects on receptor activity and trafficking and can be affected by various signalling pathways, including G-protein-coupled receptors and receptor tyrosine kinases^38,68^. Changes to spontaneous currents initiated by such signalling pathways would represent novel mechanisms through which network activity can be controlled.

In summary, we have provided a comprehensive overview of the structural determinants and modulatory mechanisms underpinning spontaneous GABA_A_R activity. Residues within the ECD (the β3 GKER motif), the M2–M3 linker (residue K279) which is important for gating, and the M3-M4 ICD (the established phosphorylation sites S408,409), all impact on the ability of β3 to support spontaneous activity. The inherent plasticity of spontaneous currents allows receptors to act not simply as leak channels, but as a highly regulated signalling pathway to control levels of neuronal activity. We have detected spontaneous currents in both synaptic and extrasynaptic receptor isoforms and spontaneous currents are therefore likely to be found throughout the brain where they could provide varying levels of inhibition. The critical importance of the β3 subunit for normal brain function is clear from knockout studies^69^ and multiple mutations of this subunit have been linked with epilepsy^70^. Exerting control over spontaneous activity in disease states may provide a novel therapeutic avenue, particularly by targeting subunit interfaces.

## Methods

### Mutagenesis

Point mutations in GABA_A_R subunits were generated using Phusion polymerase (ThermoFisher) or Q5 polymerase (New England Biolabs) in accord with manufacturers guidance. For generating chimeric subunits, restriction-free cloning was used to exchange cDNA sequences from different GABA_A_R subunit plasmids. Primers were generated and PCR conditions determined using online resources (https://www.rf-cloning.org). The primer sequences used to generate the mutant subunits are provided in Supplementary Table 2.

### Cell culture and transfection

HEK 293 cells were maintained in Dulbecco’s modified Eagle’s medium supplemented with 10% v/v heat-inactivated fetal calf serum, 100 units/ml penicillin-G and 100 µg/ml streptomycin and incubated at 37 °C in humidified 95% air/5% CO_2_. All components were obtained from Life Technologies. Cells were maintained within a passage range of P12–35. Dissociated HEK cells were plated onto 22 mm glass coverslips (VWR) coated in poly-L-lysine (Sigma). Cells were transfected >1 h later using a calcium phosphate protocol whereby a transfection solution was prepared consisting of 20 μl of 340 mM CaCl_2_, 24 μl of 2× HBS (50 mM HEPES, 280 mM NaCl, 2.8 mM Na_2_HPO_4_) and 4 μg cDNA per coverslip. All cells were transfected with equal amounts of mouse GABA_A_R subunit cDNA, and with eGFP cDNA for transfections other than those using rat δ cDNA, which included a super-ecliptic pHluorin at its N-terminus^62^. Cells were recorded from 24–48 h later.

Rat hippocampal cultures were prepared from E18 Sprague-Dawley embryos; dissociated cells were plated onto 22 mm glass coverslips coated with poly-L-ornithine (Sigma) in a plating medium consisting of minimum essential media supplemented with 5% v/v heat-inactivated fetal calf serum, 5% v/v heat-inactivated horse serum, 10 units/ml penicillin-G, 10 µg/ml streptomycin, 2 mM L-glutamine and 20 mM glucose and incubated at 37 °C in humidified 95% air/5% CO_2_. This media was replaced 2 h later with neurobasal-A media supplemented with 1% v/v B-27, 50 units/ml penicillin-G, 50 µg/ml streptomycin, 0.5% v/v glutamax and 35 mM glucose. These cells were transfected 6–8 days later using either the calcium phosphate protocol described above, or Effectene with a protocol as described by the manufacturer (Qiagen) using a total 0.8 μg of cDNA per coverslip. All cells were transfected with equal amounts of GABA_A_R subunit and eGFP cDNAs. Neurons were used for electrophysiological recording or confocal imaging 5–9 days later.

### Brain slice recording

Adolescent male rats (P21–P28) were anaesthetised by inhalation with 5% isoflurane before dispatch and isolation of the brain in accordance with the United Kingdom Animals (Scientific Procedures) Act 1986. The brain was immersed in an ice-cold slicing solution containing (mM): 85 NaCl, 2.5 KCl, 1.25 NaH_2_PO_4_, 26 NaHCO_3_, 75 sucrose, 1 CaCl_2_, 4 MgCl_2_, 25 glucose and 2 kynurenic acid, (pH 7.4 when bubbled with 95% O_2_/5% CO_2_). Horizontal (hippocampal) and coronal (thalamic) sections (all 350 µm thick) were cut using a Leica VT1200S vibratome before transfer to a holding chamber at 37 °C. The solution was slowly exchanged over 1 h with aCSF containing (mM): 125 NaCl, 2.5 KCl, 1.25 NaH_2_PO_4_, 26 NaHCO_3_, 2 CaCl_2_, 1 MgCl_2_, 25 glucose and 2 kynurenic acid (pH 7.4 when bubbled with 95% O_2_/5% CO_2_) before maintaining the slices at room temperature prior to recording.

### Whole-cell patch clamp electrophysiology

For all electrophysiological experiments, cells were visualised using a Nikon Eclipse E600FN microscope. Whole-cell currents were recorded using an Axopatch 200B amplifier and digitised using a Digidata 1322 A (Axon Instruments). Currents were acquired with Clampex ver. 10.2 and analysed with Clampfit ver. 10.7 (Axon Instruments) on a Dell Optiplex 960 M computer. Recordings were sampled at 20 kHz and filtered at 2 kHz. All recordings were performed at room temperature (20 °C). Series resistance compensation was ~70%, and cells were rejected if series resistance was too high (>20 MΩ) and if the leak current was greater than 250 pA. For prolonged recordings, cells were rejected if the series resistance changed >20% over the recording time. Drugs were applied using a fast-application Y-tube^71^.

HEK cells were recorded from using borosilicate glass electrodes with resistances ~3–5 MΩ when filled with an internal solution containing (mM): 1 MgCl_2_, 140 KCl, 11 EGTA, 10 HEPES, 1 CaCl_2_, 2 K_2_ATP, pH 7.2. Osmolarity was 300 ± 20 mOsm/L, measured using a vapour pressure osmometer (Model 5520, Wescor Inc). For experiments investigating kinase modulation, drugs were added to the internal solution to the required concentrations using stock solutions made up in DMSO, with vehicle controls performed using equivalent DMSO concentrations. Cells were perfused with a modified Kreb’s solution containing (mM): 140 NaCl, 4.7 KCl, 1.2 MgCl_2_, 2.5 CaCl_2_, 11 glucose, 5 HEPES, pH 7.4. Cells were voltage clamped at −40 mV.

Recordings from hippocampal cultures used ~3–5 MΩ electrodes filled with an internal solution consisting of (mM): 140 CsCl, 2 NaCl, 10 HEPES, 5 EGTA, 2 MgCl_2_, 0.5 CaCl_2_, 2 Na_2_ATP, 0.5 Na_2_GTP, 2 QX-314, pH 7.3. Osmolarity was 300 ± 20 mOsm/L. Cells were perfused in the same Kreb’s solution described above, except for the addition of 2 mM kynurenic acid. Cells were voltage clamped at −60 mV. For current clamp, cells were recorded using an internal electrode solution consisting of (mM): 137 potassium gluconate, 3 KCl, 10 HEPES, 5 EGTA, 0.5 CaCl_2_, 2 MgCl_2_, 2 Na_2_ATP and 0.5 Na_2_GTP, pH 7.3. Osmolarity was 300 ± 20 mOsm/L. The external solution was as described for voltage-clamp experiments and the calculated junction potential was ~15 mV. Current was injected to hold cells at −70 mV if required. For slice recordings, slices were perfused in the aCSF described above, and the internal solution was the same as that used for cultured neuronal voltage-clamp recordings.

### Single-channel recording and analysis

Single spontaneous GABA channel currents were recorded from outside-out patches of HEK cells expressing α4β3δ GABA_A_Rs and eGFP at a holding potential of −70 mV. To limit ion channel density in individual patches, HEK cells were transfected with 5 ng α4, β3, δ and eGFP cDNAs. Resulting spontaneous single-channel currents were digitised at a sampling rate of 20 kHz and filtered at 5 kHz with a 4-pole Bessel filter (24 dB per octave). A fixed time resolution of 80 μs was used as a preset. Recordings were undertaken using thick-walled borosilicate patch pipettes (6–10 MΩ) filled with a solution containing (mM): 145 CsCl, 2.5 NaCl, 10 HEPES, 1 EGTA, 4 MgATP, pH 7.3. Osmolarity was 300 ± 20 mOsm/L. Cells were perfused with an external solution consisting of (mM): 145 NaCl, 2.5 KCl, 1 CaCl_2_, 1 MgCl_2_, 10 HEPES, 10 glucose, pH 7.3. Prior control recordings were obtained using untransfected HEK cells to identify endogenous channel currents. Rarely, long open events characteristically devoid of brief closures were observed in some of these recordings, but they are easily distinguishable from the spontaneous GABA_A_R channel currents.

Channel currents were digitally low pass filtered at 1–2 kHz prior to analysis. Single-channel conductance (typically 27–30 pS) was calculated from the mean unitary channel current and the difference between the patch holding potential and GABA current reversal potential. Channel dwell states were identified using a threshold cursor method. This state transition detection method for open and shut events was used to assemble an idealised record of the digitised data. Open times affected by channel stacking were not included in the analyses and sub-conductance states were also rare and excluded from the analysis.

The analysis of the dwell times for single-channel currents was performed by fitting a mixture of exponential components to the open time distributions. These defined the mean open times and their relative proportions (area of the components) based on using a Levenberg-Marquardt non-linear least-squares routine (WinEDR ver 3.8.7, courtesy of J Dempster, University of Strathclyde).

### Quantification of spontaneous activity in HEK cells

Spontaneous activity in HEK cells was quantified by applying a saturating concentration of agonist (500 μM GABA unless otherwise stated) to elicit a maximal whole-cell current (I_Max_). Once the bath solution was exchanged and the current had fully returned to baseline (30–60 s; data were excluded if the baseline current deviated by more than 20% from its pre-application level), a saturating concentration of PTX (100 µM) was then applied until steady state was reached (3–5 s) and the current recorded (I_PTX_). Spontaneous currents (I_spont_) were quantified as a percentage of total current, as described by Eq. (1):

1 $${{{{{{\rm{I}}}}}}}_{{{{{{\rm{spont}}}}}}}( \% )=[{{{{{{\rm{I}}}}}}}_{{{{{{\rm{PTX}}}}}}}/({{{{{{\rm{I}}}}}}}_{{{{{{\rm{PTX}}}}}}}+{{{{{{\rm{I}}}}}}}_{{{{{{\rm{Max}}}}}}})]{{\,\times}}100\ldots \ldots \ldots \ldots \ldots \ldots \ldots \ldots \ldots \ldots \ldots \ldots \ldots$$

For assessment of the efficacy of allosteric modulators on spontaneous activity, compounds were briefly applied (3–5 s) to achieve steady-state currents (I_Mod_). Once the current had returned to pre-application baseline, saturating GABA and PTX concentrations were then applied. The I_Mod_ was summed with I_PTX_ (or subtracted for inhibitory modulators) and the I_spont_ calculated, as described by Eq. (2):

2 $${{{{{{\rm{I}}}}}}}_{{{{{{\rm{spont}}}}}}}( \% )=[({{{{{{\rm{I}}}}}}}_{{{{{{\rm{PTX}}}}}}}+{{{{{{\rm{I}}}}}}}_{{{{{{\rm{Mod}}}}}}})/({{{{{{\rm{I}}}}}}}_{{{{{{\rm{PTX}}}}}}}+{{{{{{\rm{I}}}}}}}_{{{{{{\rm{Max}}}}}}})]{{\,\times}}100\ldots \ldots \ldots \ldots \ldots \ldots \ldots \ldots \ldots \ldots \ldots \ldots \ldots \ldots$$

### Concentration-response curves and fitting

Increasing concentrations of GABA were applied to HEK cells transiently expressing GABA_A_Rs to generate currents. These data were plotted and fitted using a non-linear least-squares method with the Hill equation, expressed in Eq. (3):

3 $${{{{{\rm{y}}}}}}=({{{{{{\rm{y}}}}}}}_{\max }.{{{{{{\rm{A}}}}}}}^{{{{{{\rm{n}}}}}}})/({{{{{{{\rm{EC}}}}}}}_{50}}^{{{{{{\rm{n}}}}}}}+{{{{{{\rm{A}}}}}}}^{{{{{{\rm{n}}}}}}})\ldots \ldots \ldots \ldots \ldots \ldots \ldots \ldots \ldots \ldots \ldots \ldots \ldots \ldots \ldots {{{{\mathrm{.}}}}}.$$

Where A, y_max_, EC_50_ and n represent the GABA concentration, the maximum GABA current, the GABA EC_50_, and the Hill coefficient, respectively. All data were normalised to the fitted maximum response.

### shRNA and immunocytochemistry

For short hairpin RNA (shRNA)-mediated knockdown (silencing) of native β3 subunits in cultured hippocampal neurons, we used co-transfection of two pGIPZ-based constructs (Open Biosystems V2LMM_62992: TTTAAGAAATATGTGTCGG and V2LMM_65176: TTCATTGTGAACATCCATC) that selectively target rat β3. Control neurons were transfected with a pGIPZ construct containing a scrambled shRNA sequence (TCTCGCTTGGGCGAGAGTAAG). pGIPZ constructs also encoded for GFP to allow transfected cell identification.

Seven days after transfection with shRNA constructs, neurons were washed with ice-cold PBS and fixed with paraformaldehyde (4% PFA/4% sucrose/ PBS pH 7) for 5 min before extensive washing in PBS. Cells were then permeabilised using Triton X-100 (0.1%) for 5 min and then washed. Cells were incubated in a blocking solution (10% goat serum) for 30 min before brief washing and subsequent incubation for 1 h in 3% goat serum with a mouse anti-β3 primary antibody (NeuroMab, 1:500). Cells were then washed and incubated with a goat anti-mouse secondary antibody conjugated to Alexa Fluor-555 (1:500) for 1 h. After washing, coverslips were mounted on slides with Prolong Glass mounting medium (ThermoFisher Scientific) for confocal imaging.

Cells from at least three different preparations were identified through expression of GFP and were imaged using a Zeiss 510 confocal microscope with a ×40 oil objective (1.4 NA). Images were digitally captured using Zeiss LSM software (ver. 4.2) with excitation at 488 nm for GFP and 555 nm for Alexa Fluor-555.

For each neuron, sections from multiple (2–4) proximal dendrites were imaged. For quantification of neuronal β3 labelling, ImageJ (ver. 1.52p) was used to determine the mean fluorescence intensity of each dendrite. The average fluorescence intensity per cell was then determined and normalised to the average intensity of all control cells for that preparation.

### Generation of homology models

To build a homology model of the murine α4β3δ GABA_A_R based on the structure of human α1β3γ2 (PDB template: 6I53), we created an alignment file, for use by Modeller ver. 9.19, in a pentameric format. This required optimal alignments of murine α4 versus human α1, murine β3 versus human β3, and murine δ versus human γ2, using the EMBL-EBI multiple sequence alignment webtool ClustalW2.

Aligning all murine and human GABA_A_R α(1–6) subunits revealed the expected high degree of sequence homology (Supplementary Table 3). Template (human α1) from PDB 6I53, is shown highlighted in yellow with corresponding primary sequence for murine α4 shown in green highlight. The intracellular region between M3 and M4 that is not resolved in 6I53 (red highlight) was excluded in the Modeller alignment file. A similar approach was adopted for aligning murine and human GABA_A_R β(1–3) subunits. Murine and human β3 primary sequences are identical in the regions included in the model (Supplementary Table 4). The most difficult alignment involves that for murine δ and human γ2. We therefore aligned all GABA_A_R non α, non β subunits that could fill the fifth position in the αβX heteropentamer, notably: γ1-3 (GBRG1-3), δ (GBRD), ε (GBRE), θ (GBRT), π (GBRP). This approach provided confidence in the alignments of murine δ and human γ2 for the regions included in the model (Supplementary Table 5). Using all three alignments, a pentameric alignment file for Modeller 9.19 was constructed and used to generate 50 homology models of the α4β3δ GABA_A_R.

The model quality estimation webtool for membrane proteins, QMEANBrane (http://swissmodel.expasy.org/qmean/), was used to rank all 50 models to obtain the most appropriate structures, which were subsequently processed in SCRWL4^72^ to optimise side-chain configurations. Further model optimisation was performed using MOLProbity (http://molprobity.biochem.duke.edu/) where Asn/Gln/His residues were flipped if clear evidence was provided for protonation. The α4β3δ GABA_A_R homology model was then structure minimised in Chimera before final runs through MOLProbity once more, where protein geometry scores showed successful iterative model optimisation leading to the final homology model. All structural images were visualised and rendered using the molecular graphics systems, PyMOL (Schrodinger) and UCSF Chimera.

### Statistical analysis and reproducibility

Data are represented as the mean ± standard error of the mean (SEM). Individual data points are included within figures. Data were analysed and statistical tests performed using Origin 2019 (OriginLab). All datasets were tested for normality using the Kolmogorov–Smirnov test with Lilliefors correction. If data were normally distributed, two-sided unpaired *t*-tests, paired *t*-tests and ANOVA with Tukey’s post-hoc tests were used as indicated. For non-Gaussian distributed data, two-sided Mann–Whitney, paired samples Wilcoxon and Kruskal–Wallis with Dunn’s post-hoc tests were used. A *P* value of <0.05 was set as the level of significance and denoted by *. When *P* < 0.01 or *P* < 0.001, this is represented by ** and ***, respectively. The n numbers in the figure legends signify how many times each experiment was repeated independently to ensure reproducible results.

### Reporting summary

Further information on experimental design is available in the Nature Research Reporting Summary linked to this paper.

## Supplementary information

Supplementary Information

Reporting summary

## Data Availability

The datasets generated and analysed during this study are included in this published article and in the associated supplementary information files﻿. Any other data associated with this paper are available from the corresponding author upon reasonable request. Source data are provided with this paper.

## Source data

Source Data
