# Supplementary material for: Structural determinants and regulation of spontaneous activity in GABAA receptors
Source: Nat Commun. 2021 Sep 15;12:5457. doi: 10.1038/s41467-021-25633-0 (PMC8443696; doi:10.1038/s41467-021-25633-0)
Supplement: Supplementary file 1 — Supplementary Information [file 41467_2021_25633_MOESM1_ESM.pdf]

Supplementary Figure 1

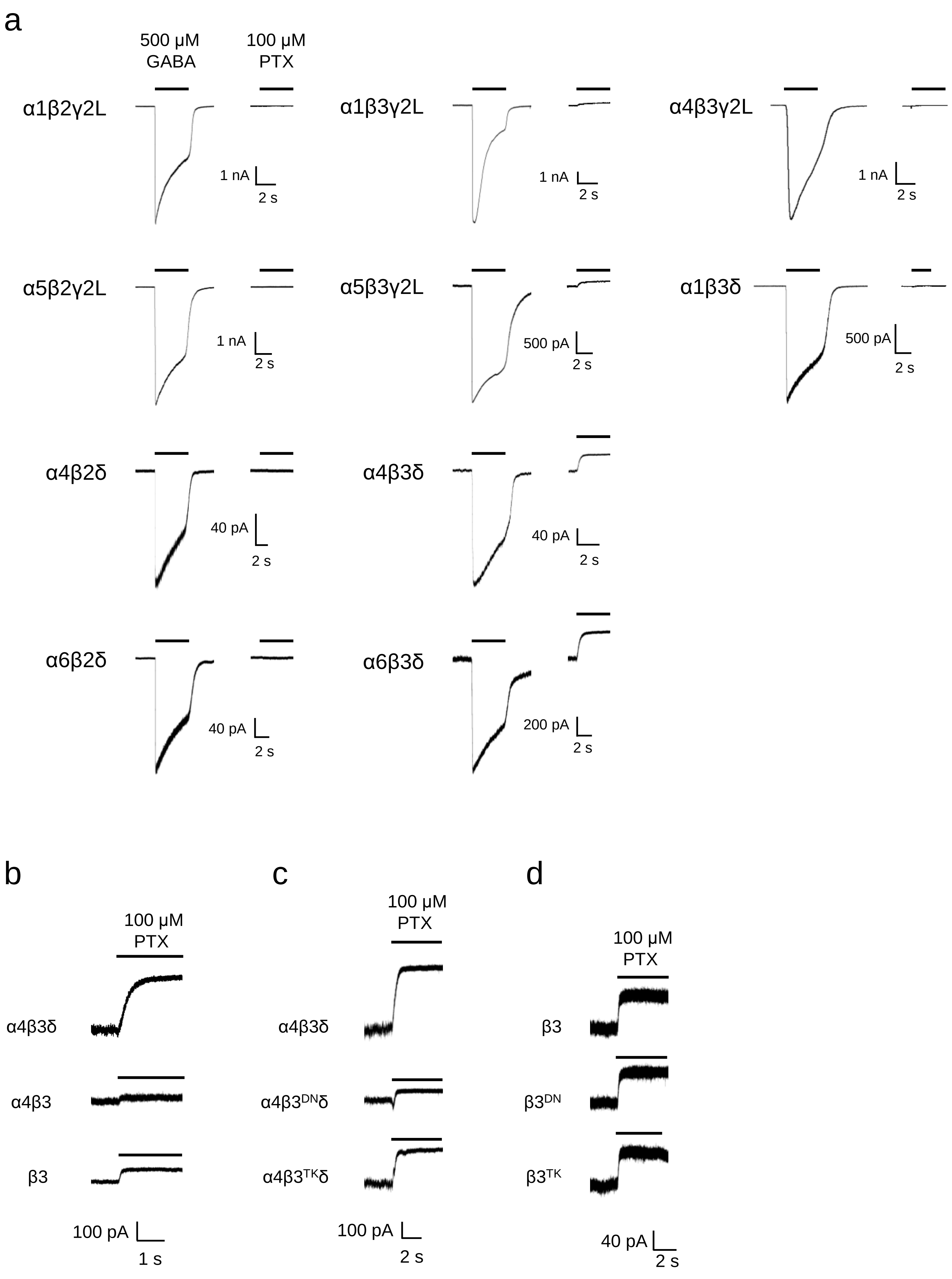

Supplementary Figure 1: Voltage clamp recordings from HEK cells expressing wild-type and mutant recombinant $GABA_A$ receptors. **a**, Recordings from HEK cells expressing recombinant triheteromeric receptors showing the effect of GABA (500 μM) and PTX (100 μM). **b-d**, Recordings of spontaneous currents for tri- and diheteromeric and homomeric GABA receptors, including wild-type and chimeric subunits.

Supplementary Figure 2

a

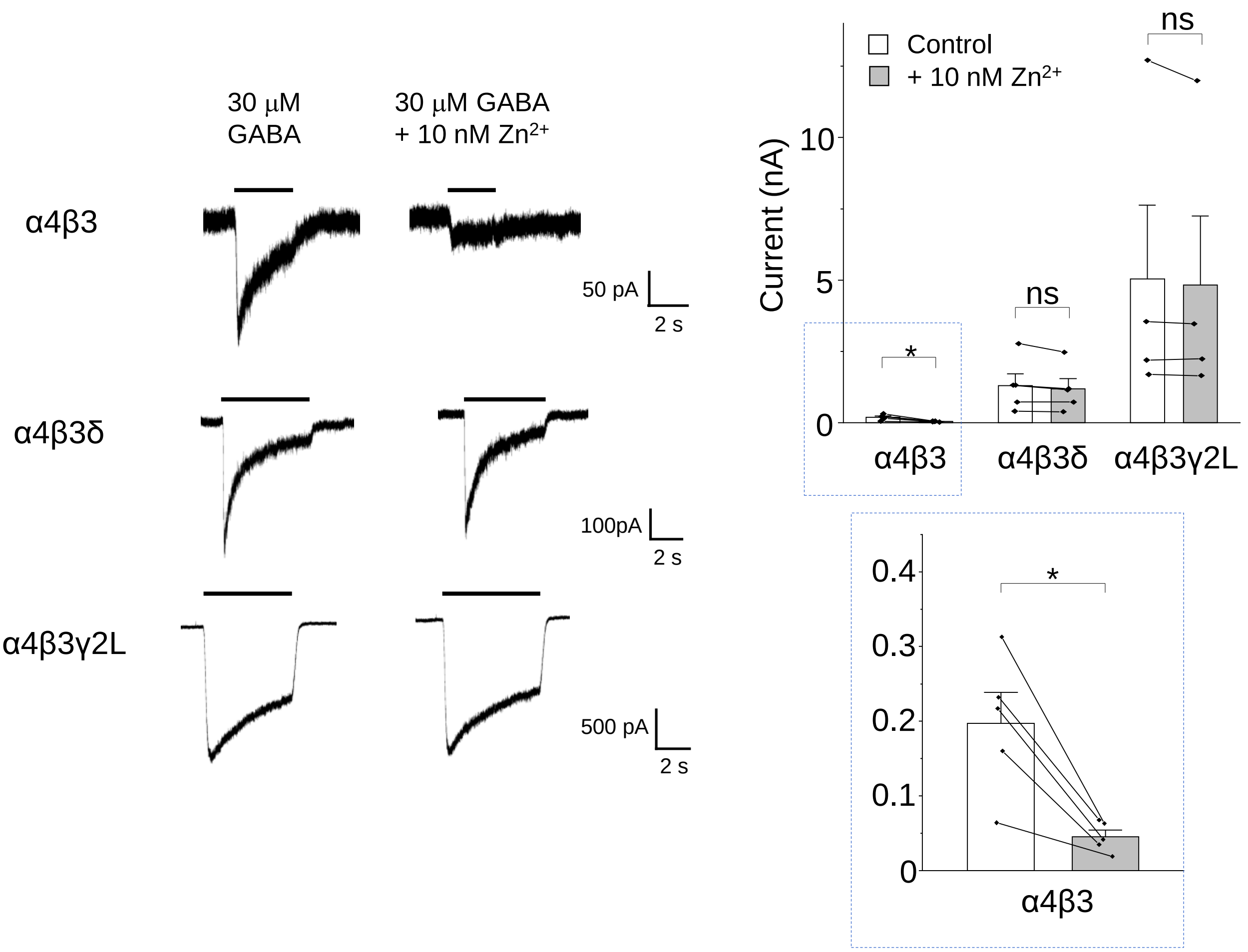

b

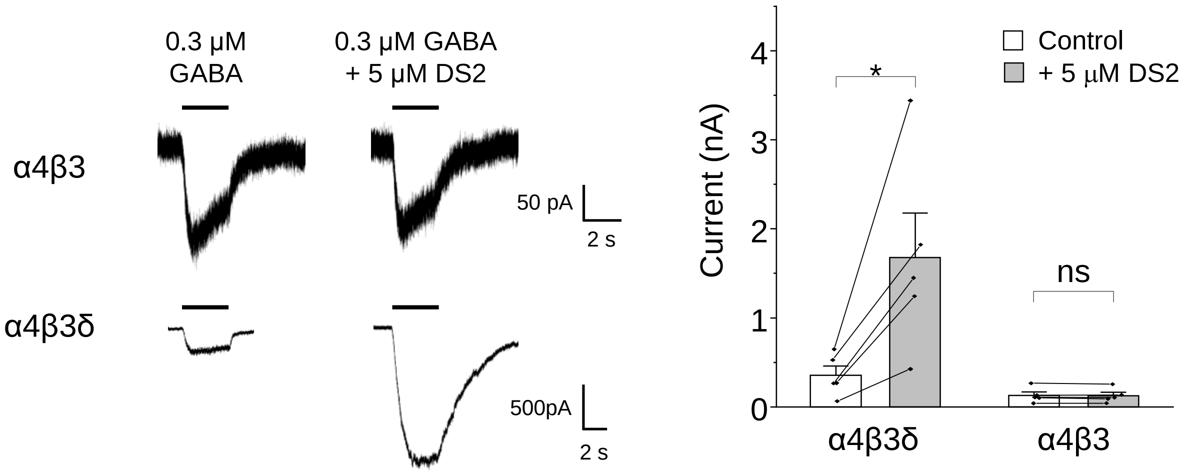

Supplementary Figure 2: HEK cells transfected with three subunit cDNAs express predominantly triheteromeric receptors. **a**, Left: recordings of diheteromeric and triheteromeric receptor responses to GABA (30 μM) alone and in the presence of Zn^{2+} (10 nM). Right: Zn^{2+} was a potent antagonist at diheteromeric α4β3 receptors (*P=0.011; n=5) but had insignificant effects at triheteromeric α4β3δ (P=0.098; n=5) and α4β3γ2L (P=0.33; n=4) receptors, indicating the majority of current recorded from these cells represented triheteromeric receptors. Inset shows expanded view of the current change at the α4β3 receptor. Comparisons were made using two-sided paired t-tests. **b**, Left: recordings from α4β3 and α4β3δ expressing HEK cells during application of GABA (0.3 μM) and with the δ-selective potentiator DS2 (5 μM). Right: DS2 significantly enhanced the current in cells expressing α, β and δ subunits (*P=0.030; n=5) but had no effect on cells expressing α and β subunits (P=0.34; n=5). This confirms that expressed triheteromeric receptors incorporated the δ subunit. Comparisons were made using two-sided paired t-tests. Data are presented as mean values ± SEM. *P<0.05; ns - no significance. Source data are provided as a Source Data file for Supplementary Fig. 2.

Supplementary Figure 3

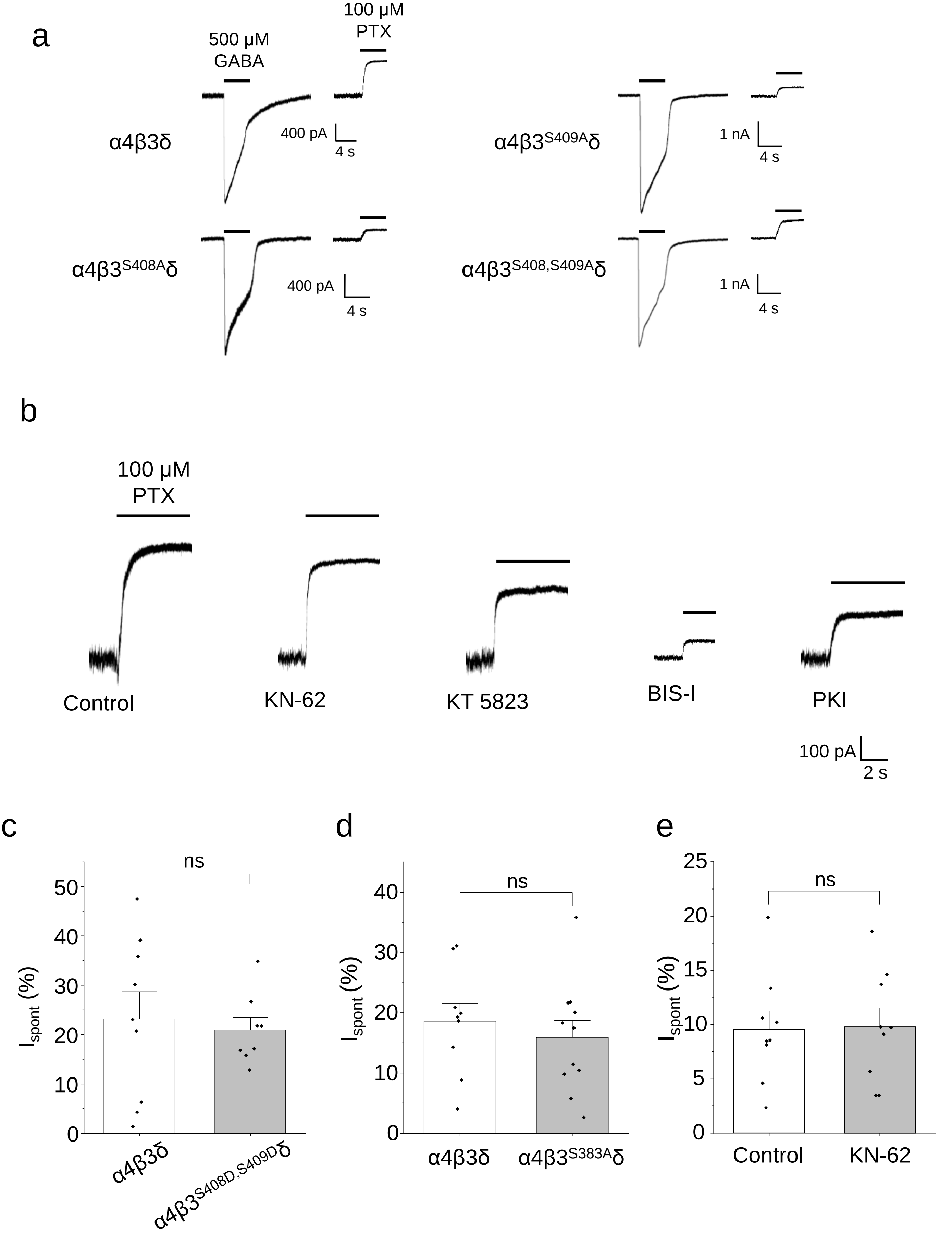

Supplementary Figure 3: Modulation of β3 subunit phosphorylation affects spontaneous activity. a, Current recordings of wild-type α4β3δ and mutant-containing β3 S408A and S409A subunits in HEK cells. Removing b3 phosphorylation sites reduced I_{spont}. **b**, Representative I_{PTX} recordings of α4β3δ receptors exposed to various selective kinase inhibitors applied intracellularly via the patch electrode. I_{PTX} was measured 2 mins after whole-cell breakthrough. **c**, I_{spont} did not differ between α4β3δ and the phosphomimetic $α4β3^{S408D,S409D}δ$ receptors suggesting receptors are maximally phosphorylated at these residues under control conditions (P=0.72; two-sided unpaired t-test; α4β3δ: n=9; $α4β3^{S408D,S409D}δ$: n=8). **d**, I_{spont} of α4β3δ and $α4β3^{S383A}δ$, which lacks the CaMKII phosphorylation site within the large intracellular loop. The lack of effect of S383A suggested it has no role in spontaneous activity (P=0.26; two-sided unpaired t-test; α4β3δ: n=8; $α4β3^{S383A}δ$: n=10). **e**, The I_{spont} of α4β3δ receptors exposed to the selective CaMKII inhibitor KN-62 (3 μM) applied intracellularly via the patch electrode. I_{PTX} was measured 2 mins after whole-cell breakthrough. There was no effect compared to the vehicle control, further suggesting CaMKII has little direct impact on spontaneous activity (P=0.92; two-sided unpaired t-test; n=9). Data are presented as mean values ± SEM. ns - no significance. Source data are provided as a Source Data file for Supplementary Fig. 3.

Supplementary Figure 4

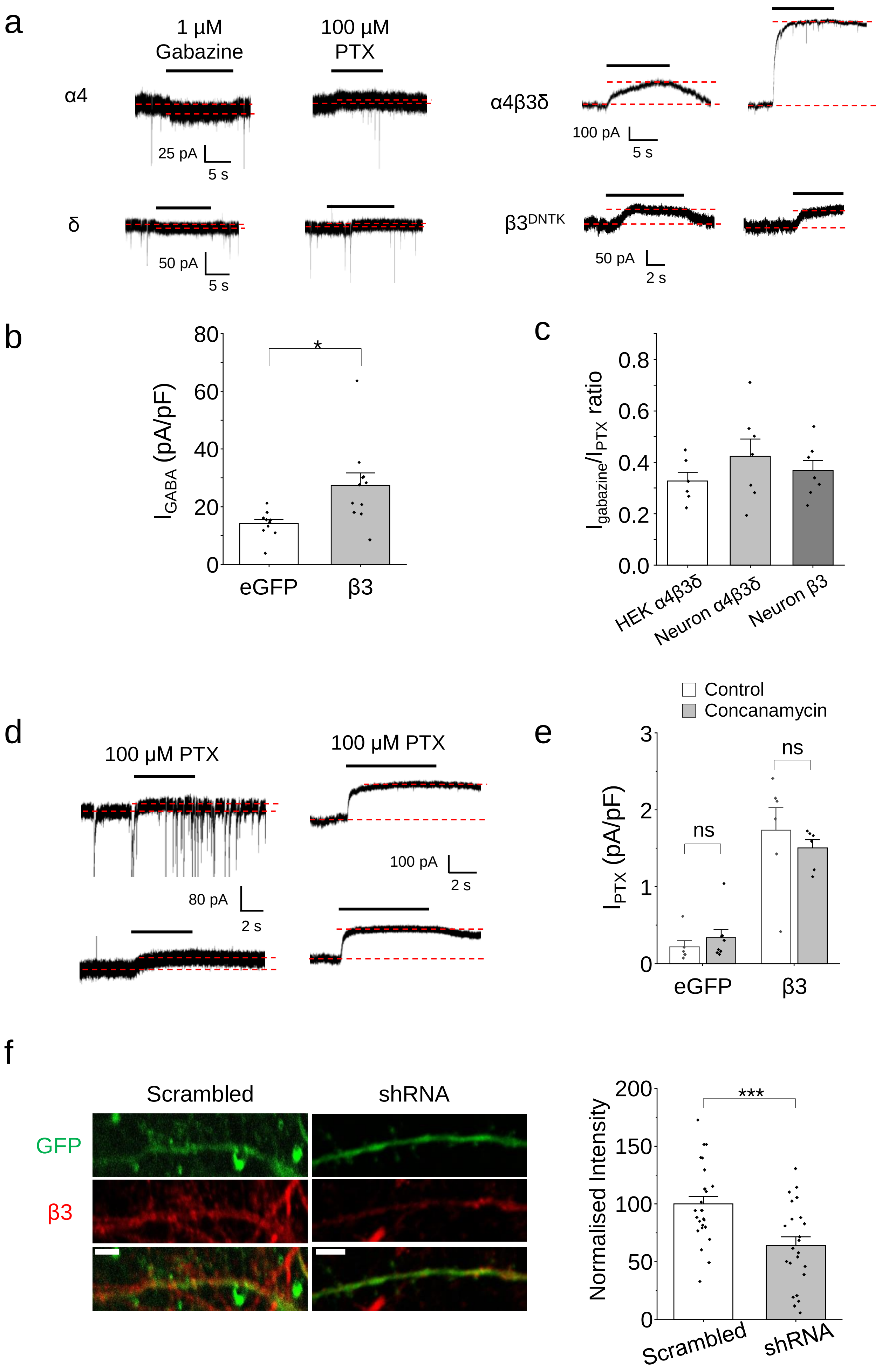

Supplementary Figure 4: Spontaneous current activity in hippocampal neurons depends on β3-containing heteromeric $GABA_A$Rs. a, Recordings from neurons transfected with various $GABA_A$R cDNAs exposed to gabazine (1 μM) and PTX (100 μM). **b**, Current density evoked by 1 μM GABA in neurons transfected with β3 cDNA compared to control eGFP (*P=0.012; two-sided unpaired t-test; eGFP: n=10; β3: n=11). The greater response to GABA indicates greater expression of heteromeric receptors. **c**, Ratio of currents caused by gabazine (1 μM) and PTX (100 μM) for neurons and HEK cells transfected with α4, β3 and δ cDNAs and neurons transfected only with β3. The lack of difference suggests similar ratios of heteromeric α4β3δ and homomeric β3 receptors are expressed in these transfected cells (P≥0.39; one-way ANOVA with Tukey's post hoc test; HEK α4β3δ: n=6; neuron α4β3δ: n=7; neuron β3: n=7). **d**, Hippocampal neurons transfected with eGFP alone or with β3 cDNA were incubated with concanamycin A (500 nM) for 1-2 hrs to prevent vesicular replenishment and subsequent synaptic GABA release. Recordings of eGFP- (left) and β3-expressing neurons (right) incubated under control conditions (upper panels) or with concanamycin A (lower panels) during PTX application are shown. **e**, No difference was observed in spontaneous tonic current density, determined by PTX (100 μM), between neurons treated with concanamycin A and with a DMSO control for either eGFP (P=0.18; two-sided Mann-Whitney test; control: n=6; concanamycin: n=8) or β3-transfected (P=0.49; two-way unpaired t-test; n= 6) neurons. **f**, Neurons from 3 separate cultures were fixed, permeabilised and immunostained with selective anti-β3 antibodies and the level of subunit expression determined. Left: images of proximal dendrites of neurons transfected with either scrambled control or shRNA for β3 subunits. Scale bars represent 5 μm. Right: neurons expressing shRNA displayed 64% fluorescence intensity compared to that of neurons transfected with the scrambled control (***P=0.00066; two-sided unpaired t-test; scrambled: n=26; shRNA: n=23). Data are presented as mean values ± SEM. *P<0.05; ***P<0.001; ns - no significance. Source data are provided as a Source Data file for Supplementary Fig. 4.

Supplementary Table 1: Spontaneous activity of wild-type and mutant $GABA_A$Rs.

Receptor Constructs: Wild-type	I_{Spont} (%)
α1β2γ2L	0.13 ± 0.04, n = 7
α1β3γ2L	1.5 ± 0.3, n = 6
α1β3δ	0.49 ± 0.10, n = 7
α4β1δ	0.16 ± 0.06, n = 8
α4β2δ	0.02 ± 0.02, n = 6
α4β3δ	19 ± 2, n = 27
α4β3γ2L	0.67 ± 0.11, n = 10
α5β2γ2L	0.27 ± 0.15, n = 7
α5β3γ2L	4.0 ± 1.1, n = 9
α6β2δ	0.17 ± 0.13, n = 7
α6β3δ	15 ± 4, n = 21
**Structural Mutants**	**I_{Spont} (%)**
$α4β2^{β3(ECD)}δ$	23 ± 5, n = 9
$α4β3^{β2(ECD)}δ$	0.41 ± 0.18, n = 8
$α4β2^{GKER}δ$	19 ± 4, n = 8
$α4β3^{DNTK}δ$	0.9 ± 0.6, n = 7
$α4β3^{DN}δ$	4.9 ± 1.2, n = 8
$α4β3^{TK}δ$	8.1 ± 3.3, n = 10
$α4^{α1(ECD)}β3δ$	11 ± 1, n = 12
$α4^{α1(loop\ C)}β3δ$	28 ± 3, n = 8
$α4^{R100H}β3δ$	8.2 ± 2.5, n = 7
$α4β3δ^{γ2L(ECD)}$	6.3 ± 1.3, n = 8
$α4β3δ^{γ2L(loop\ C)}$	10 ± 2, n = 8
$α4β3^{K279T}γ2L$	74 ± 5, n = 6
**Phosphorylation Mutants**	**I_{spont} (%)**
$α4β3^{S408A}δ$	8.0 ± 1.7, n = 16
$α4β3^{S409A}δ$	4.4 ± 1.0, n = 12
$α4β3^{S408A,\ S409A}δ$	14 ± 2, n = 17
$α4β3^{S408D,\ S409D}δ$	21 ± 3, n = 8
$α4β3^{S383A}δ$	16 ± 3, n = 10

Supplementary Table 1: I_{spont} of a variety of recombinant $GABA_A$R constructs are shown. Values are mean ± SEM for n cells.

Supplementary Table 2: Primer sequences used for mutagenesis of $GABA_A$R subunits.

Subunit	Forward primer	Reverse primer
$\alpha 4^{\alpha 1(ECD)}$	CTCGGTTCTATCGATTGAATTCGCCACCATGAAGAAAAGT CGGGGTCTCTC	CTGTCATGATGCATGGGATATACGTCTGAATCATAAAGCCAATTTTTC TCTTCAAGTGGAAG
$\alpha 4^{\alpha 1(loop\ C)}$	CATTGGGCAGACTGTATCAAGCGAGATTGTTCAGTCCAGT ACTGGAGAA	TCTGAATCATAAAGTAGCCCATCTTCCGTCTCAAGTGGAAGTGAGTC GTCATA
$\alpha 4^{R100H}$	TACTTTCTTCCATAATGGAAAGAAATCTGTCTC	TCAGGGGTCCAAACTTTG
$\alpha 4^{Q246M}$	GATTCTTTCTATGGTTTCCTTCTGGATAAAC	ACTGTCATGATGCATGGG
$\beta 2^{\beta 3(ECD)}$	TCCTCAATCCCACCAGAACCTAAAGGGATGTGGGGCTTTG CGGGAG	CAGAATTGATGGCATGTATGTCTGCAGGATGAAGTACCCGATATTTCT CTTCAACCGAA
$\beta 2^{GKER}$	ACATTGAGTTTTACTGGCGCGGCGGGGACAAGGCTG	GAGAACTGAGGAAGCTCAATCCTTTCCACGCCAGTGA
$\beta 3^{\beta 2(ECD)}$	CCGGGGCGCGGCGAAGGGATGTGGAGAGTCCGGAAAAG	ATTGAGGGCATATACGTCTGCAGAATGAAGTAGCCAATGTTCCTTTTC AGC
$\beta 3^{DNTK}$	ACATTGAATTTTACTGGCGTGGCGATGACAATGCAG	GAGAACTGTGGGAGCTCGATCTTTGTCACTCCTGTGA
$\beta 3^{DN}$	GACAATGCTGTCACTGGCGT	ATCGCCACGCCAGTAAAATT
$\beta 3^{TK}$	AAAGATCGAGCTCCCACAGTTCTC	GTCACGCCAGTGACAGCCTT
$\beta 3^{K279T}$	AAATTCCCTATGTCACAGCCATCGACATGTACCTG	TGGGTAGAGTCTCCCGAAGGTGAGTGTTGATGGTT
$\beta 3^{S383A}$	TAGGAAACAGGCCATGCCCAAG	TACTGGATTCCTGAGTTG
$\beta 3^{S408A}$	ACGGAGGAGGGCTTCACAGCT	AGGTGGGTCTTCTTGTGCG
$\beta 3^{S409A}$	GAGGAGGTCTGCACAGCTCAA	CGTAGGTGGGTCTTCTTG
$\beta 3^{S408A,S409A}$	ACGGAGGAGGGCTGCACAGCTCAA	AGGTGGGTCTTCTTGTGCG
$\beta 3^{S408D,S409D}$	CAGCTCAAAATCAAAATCCC	GTCATCCCTCCTCCGTAGGT
$\delta^{\gamma 2L(ECD)}$	GCACCTCGGTTCTATCGATTGAATTCGCCATGAGTTCGCC AAATACATGGAGC	AGAGGGCATGTAAGACTGGATGATGTAGACGCCCATTCTTCTGCTCA GATCG
$\delta^{\gamma 2L(loop\ C)}$	CCAGTTCACTATCACCAGTTACCGCTTCACAACTGAAGTA GTGAAGACAACTTCTGGTGACTAT	CGGTTCCTCCGAAGCTGGAAGTGTAAAGACATCACCACATAGTCACC AGAAGTTGTCTTCACTA

Supplementary Table 2: Nucleotide sequences of the primers used in PCR for the generation of mutant receptor subunits, denoted 5’ to 3’. Mutant subunits are either point mutants or, if denoted by a subunit followed by a subunit area in parentheses, chimeric subunits. Residue numbering is based upon the mature murine protein, or as previously defined in the literature.

Supplementary Table 3: ClustalW2 alignment of murine and human α1-6 subunits.

```
sp|P14867|GBRA1_HUMAN  MRKSPGLSDC------LWAWIL-L-LSTLTGRSY----------------
sp|P16305|GBRA6_MOUSE  MVLL----------------LPWLFIILWLENAQA---------------
sp|P26048|GBRA2_MOUSE  MKTKLST-CN------VWSLLLVL-LVW-DPVRL----------------
sp|P26049|GBRA3_MOUSE  MIITQMWHFY------VTRVVLLLLISIL-PGTTSQGESRRQEPGDFVKQ
sp|P31644|GBRA5_HUMAN  MDNGMFSGFI------MIKNLLLFCISMNLSSHFGFSQ------------
sp|P34903|GBRA3_HUMAN  MIITQTSHCY------MTSLGILFLINIL-PGTTGQGESRRQEPGDFVKQ
sp|P47869|GBRA2_HUMAN  MKTKLNI-YN------MQFLLFVF-LVW-DPARL----------------
sp|P48169|GBRA4_HUMAN  MVSAKKVPAIALSAGVSFALLRFLCLAVCLNESPG---------------
sp|P62812|GBRA1_MOUSE  MKKSRGLSDY------LWAWTL-I-LSTLSGRSY----------------
sp|Q16445|GBRA6_HUMAN  MASS----------------LPWLCIILWLENALG---------------
sp|Q8BHJ7|GBRA5_MOUSE  MDNGMLSRFI------MTQTLLVFCISMTLSSHFGFSQ------------
sp|Q9D6F4|GBRA4_MOUSE  MVSVQKVPAIALCSGVSLALLHFLCLAACLNESPG---------------
                       *                      : :

sp|P14867|GBRA1_HUMAN  --------GQPSLQDELKDNTTVFTRILDRLLDGYDNRLRPGLGERVTEV
sp|P16305|GBRA6_MOUSE  -----------QLEDEGNFYSENVSRILDNLLEGYDNRLRPGFGGAVTEV
sp|P26048|GBRA2_MOUSE  -------VLANIQEDEAKNNITIFTRILDRLLDGYDNRLRPGLGDSITEV
sp|P26049|GBRA3_MOUSE  DIGGLSPKHAPDIPDDSTDNITIFTRILDRLLDGYDNRLRPGLGDAVTEV
sp|P31644|GBRA5_HUMAN  -------MPTSSVKDETNDNITIFTRILDGLLDGYDNRLRPGLGERITQV
sp|P34903|GBRA3_HUMAN  DIGGLSPKHAPDIPDDSTDNITIFTRILDRLLDGYDNRLRPGLGDAVTEV
sp|P47869|GBRA2_HUMAN  -------VLANIQEDEAKNNITIFTRILDRLLDGYDNRLRPGLGDSITEV
sp|P48169|GBRA4_HUMAN  -----------QNQKEEKLCTENFTRILDSLLDGYDNRLRPGFGGPVTEV
sp|P62812|GBRA1_MOUSE  ---------GQP-SQDELKDNTTVFTRILDRLLDGYDNRLRPGLGERVTEV
sp|Q16445|GBRA6_HUMAN  -----------KLEVEGNFYSENVSRILDNLLEGYDNRLRPGFGGAVTEV
sp|Q8BHJ7|GBRA5_MOUSE  -------MPTSSVQDETNDNITIFTRILDGLLDGYDNRLRPGLGERITQV
sp|Q9D6F4|GBRA4_MOUSE  -----------QNSKDEKLCPENFTRILDSLLDGYDNRLRPGFGGPVTEV
                                      : .     .:**** **:*********:*  :*:*

sp|P14867|GBRA1_HUMAN  KTDIFVTSFGPVSDHDMEYTIDVFFRQSWKDERLKFKGPMTVLRLNNLMA
sp|P16305|GBRA6_MOUSE  KTDIYVTSFGPVSDVEMEYTMDVFFRQTWTDERLKFKGPAEILSLNNLMV
sp|P26048|GBRA2_MOUSE  FTNIYVTSFGPVSDTDMEYTIDVFFRQKWKDERLKFKGPMNILRLNNLMA
sp|P26049|GBRA3_MOUSE  KTDIYVTSFGPVSDTDMEYTIDVFFRQTWHDERLKFDGPMKILPLNNLLA
sp|P31644|GBRA5_HUMAN  RTDIYVTSFGPVSDTEMEYTIDVFFRQSWKDERLRFKGPMQRLPLNNLLA
sp|P34903|GBRA3_HUMAN  KTDIYVTSFGPVSDTDMEYTIDVFFRQTWHDERLKFDGPMKILPLNNLLA
sp|P47869|GBRA2_HUMAN  FTNIYVTSFGPVSDTDMEYTIDVFFRQKWKDERLKFKGPMNILRLNNLMA
sp|P48169|GBRA4_HUMAN  KTDIYVTSFGPVSDVEMEYTMDVFFRQTWIDKRLKYDGPIEILRLNNMMV
sp|P62812|GBRA1_MOUSE  KTDIFVTSFGPVSDHDMEYTIDVFFRQSWKDERLKFKGPMTVLRLNNLMA
sp|Q16445|GBRA6_HUMAN  KTDIYVTSFGPVSDVEMEYTMDVFFRQTWTDERLKFGGPTEILSLNNLMV
sp|Q8BHJ7|GBRA5_MOUSE  RTDIYVTSFGPVSDTEMEYTIDVFFRQSWKDERLRFKGPMQRLPLNNLLA
sp|Q9D6F4|GBRA4_MOUSE  KTDIYVTSFGPVSDVEMEYTMDVFFRQTWIDKRLKYDGPIEILRLNNMMV
                        *:*:********* :****:******.* *:**:: **   * ***::.

sp|P14867|GBRA1_HUMAN  SKIWTPDTFFHNGKKSVAHNMTMPNKLLRITEDGTLLYTMRLTVRAECPM
sp|P16305|GBRA6_MOUSE  SKIWTPDTFFRNGKKSIAHNMTTPNKLFRLMQNGTILYTMRLTINADCPM
sp|P26048|GBRA2_MOUSE  SKIWTPDTFFHNGKKSVAHNMTMPNKLLRIQDDGTLLYTMRLTVQAECPM
sp|P26049|GBRA3_MOUSE  SKIWTPDTFFHNGKKSVAHNMTTPNKLLRLVDNGTLLYTMRLTIHAECPM
sp|P31644|GBRA5_HUMAN  SKIWTPDTFFHNGKKSIAHNMTTPNKLLRLEDDGTLLYTMRLTISAECPM
sp|P34903|GBRA3_HUMAN  SKIWTPDTFFHNGKKSVAHNMTTPNKLLRLVDNGTLLYTMRLTIHAECPM
sp|P47869|GBRA2_HUMAN  SKIWTPDTFFHNGKKSVAHNMTMPNKLLRIQDDGTLLYTMRLTVQAECPM
sp|P48169|GBRA4_HUMAN  TKVWTPDTFFRNGKKSVSHNMTAPNKLFRIMRNGTILYTMRLTISAECPM
sp|P62812|GBRA1_MOUSE  SKIWTPDTFFHNGKKSVAHNMTMPNKLLRITEDGTLLYTMRLTVRAECPM
sp|Q16445|GBRA6_HUMAN  SKIWTPDTFFRNGKKSIAHNMTTPNKLFRIMQNGTILYTMRLTINADCPM
sp|Q8BHJ7|GBRA5_MOUSE  SKIWTPDTFFHNGKKSIAHNMTTPNKLLRLEDDGTLLYTMRLTISAECPM
sp|Q9D6F4|GBRA4_MOUSE  TKVWTPDTFFRNGKKSVSHNMTAPNKLFRIMRNGTILYTMRLTISAECPM
                       :*:*******:*****::**** ****:*:  :**:*******: *:***
```

```
sp|P14867|GBRA1_HUMAN   HLEDFPMDAHACPLKFGSYAYTRAEVVYEWTREPARSVVVAEDGSRLNQY
sp|P16305|GBRA6_MOUSE   RLVNFPMDGHACPLKFGSYAYPKTEIIYTWKKGPLYSVEVPEESSSLLQY
sp|P26048|GBRA2_MOUSE   HLEDFPMDAHSCPLKFGSYAYTTSEVTYIWTYNASDSVQVAPDGSRLNQY
sp|P26049|GBRA3_MOUSE   HLEDFPMDVHACPLKFGSYAYTKAEVIYSWTLGKNKSVEVAQDGSRLNQY
sp|P31644|GBRA5_HUMAN   QLEDFPMDAHACPLKFGSYAYPNSEVVYVWTNGSTKSVVVAEDGSRLNQY
sp|P34903|GBRA3_HUMAN   HLEDFPMDVHACPLKFGSYAYTTAEVVYSWTLGKNKSVEVAQDGSRLNQY
sp|P47869|GBRA2_HUMAN   HLEDFPMDAHSCPLKFGSYAYTTSEVTYIWTYNASDSVQVAPDGSRLNQY
sp|P48169|GBRA4_HUMAN   RLVDFPMDGHACPLKFGSYAYPKSEMIYTWTKGPEKSVEVPKESSSLVQY
sp|P62812|GBRA1_MOUSE   HLEDFPMDAHACPLKFGSYAYTRAEVVYEWTREPARSVVVAEDGSRLNQY
sp|Q16445|GBRA6_HUMAN   RLVNFPMDGHACPLKFGSYAYPKSEIIYTWKKGPLYSVEVPEESSSLLQY
sp|Q8BHJ7|GBRA5_MOUSE   QLEDFPMDAHACPLKFGSYAYPNSEVVYVWTNGSTKSVVVAEDGSRLNQY
sp|Q9D6F4|GBRA4_MOUSE   RLVDFPMDGHACPLKFGSYAYPKSEMIYTWTKGPEKSVEVPKESSSLVQY
                        :* :**** *:**********. :*: * *.     ** *. :.* * **

sp|P14867|GBRA1_HUMAN   DLLGQTVDSGIVQSSTGEYVVMTTHFHLKRKIGYFVIQTYLPCIMTVILS
sp|P16305|GBRA6_MOUSE   DLIGQTVSSETIKSNTGEYVIMTVYFHLQRKMGYFMIQIYTPCIMTVILS
sp|P26048|GBRA2_MOUSE   DLLGQSIGKETIKSSTGEYTVMTAHFHLKRKIGYFVIQTYLPCIMTVILS
sp|P26049|GBRA3_MOUSE   DLLGHVVGTEIIRSSTGEYVVMTTHFHLKRKIGYFVIQTYLPCIMTVILS
sp|P31644|GBRA5_HUMAN   HLMGQTVGTENISTSTGEYTIMTAHFHLKRKIGYFVIQTYLPCIMTVILS
sp|P34903|GBRA3_HUMAN   DLLGHVVGTEIIRSSTGEYVVMTTHFHLKRKIGYFVIQTYLPCIMTVILS
sp|P47869|GBRA2_HUMAN   DLLGQSIGKETIKSSTGEYTVMTAHFHLKRKIGYFVIQTYLPCIMTVILS
sp|P48169|GBRA4_HUMAN   DLIGQTVSSETIKSITGEYIVMTVYFHLRRKMGYFMIQTYIPCIMTVILS
sp|P62812|GBRA1_MOUSE   DLLGQTVDSGIVQSSTGEYVVMTTHFHLKRKIGYFVIQTYLPCIMTVILS
sp|Q16445|GBRA6_HUMAN   DLIGQTVSSETIKSNTGEYVIMTVYFHLQRKMGYFMIQIYTPCIMTVILS
sp|Q8BHJ7|GBRA5_MOUSE   HLMGQTVGTENISTSTGEYTIMTAHFHLKRKIGYFVIQTYLPCIMTVILS
sp|Q9D6F4|GBRA4_MOUSE   DLIGQTVSSETIKSITGEYIVMTVYFHLRRKMGYFMIQTYIPCIMTVILS
                        .*:*: :..  : : **** :**.:***:**:***:** * *********

sp|P14867|GBRA1_HUMAN   QVSFWLNRESVPARTVFGVTTVLTMTTLSISARNSLPKVAYATAMDWFIA
sp|P16305|GBRA6_MOUSE   QVSFWINKESVPARTVFGITTVLTMTTLSISARHSLPKVSYATAMDWFIA
sp|P26048|GBRA2_MOUSE   QVSFWLNRESVPARTVFGVTTVLTMTTLSISARNSLPKVAYATAMDWFIA
sp|P26049|GBRA3_MOUSE   QVSFWLNRESVPARTVFGVTTVLTMTTLSISARNSLPKVAYATAMDWFIA
sp|P31644|GBRA5_HUMAN   QVSFWLNRESVPARTVFGVTTVLTMTTLSISARNSLPKVAYATAMDWFIA
sp|P34903|GBRA3_HUMAN   QVSFWLNRESVPARTVFGVTTVLTMTTLSISARNSLPKVAYATAMDWFIA
sp|P47869|GBRA2_HUMAN   QVSFWLNRESVPARTVFGVTTVLTMTTLSISARNSLPKVAYATAMDWFIA
sp|P48169|GBRA4_HUMAN   QVSFWINKESVPARTVFGITTVLTMTTLSISARHSLPKVSYATAMDWFIA
sp|P62812|GBRA1_MOUSE   QVSFWLNRESVPARTVFGVTTVLTMTTLSISARNSLPKVAYATAMDWFIA
sp|Q16445|GBRA6_HUMAN   QVSFWINKESVPARTVFGITTVLTMTTLSISARHSLPKVSYATAMDWFIA
sp|Q8BHJ7|GBRA5_MOUSE   QVSFWLNRESVPARTVFGVTTVLTMTTLSISARNSLPKVAYATAMDWFIA
sp|Q9D6F4|GBRA4_MOUSE   QVSFWINKESVPARTVFGITTVLTMTTLSISARHSLPKVSYATAMDWFIA
                        *****:*:**********:**************:*****:**********

sp|P14867|GBRA1_HUMAN   VCYAFVFSALIEFATVNYFTKRGYAWDGKSVVPE-KPKKVKDP-------
sp|P16305|GBRA6_MOUSE   VCFAFVFSALIEFAAVNYFTNLQSQKAERQAQTA-AT----PPVAKSKAS
sp|P26048|GBRA2_MOUSE   VCYAFVFSALIEFATVNYFTKRGWAWDGKSVVND-KKKEKG-S-------
sp|P26049|GBRA3_MOUSE   VCYAFVFSALIEFATVNYFTKRSWAWEGKKVPEALEMKKKTPAA------
sp|P31644|GBRA5_HUMAN   VCYAFVFSALIEFATVNYFTKRGWAWDGKKALEAAKIKKKR-EV------
sp|P34903|GBRA3_HUMAN   VCYAFVFSALIEFATVNYFTKRSWAWEGKKVPEALEMKKKTPAA------
sp|P47869|GBRA2_HUMAN   VCYAFVFSALIEFATVNYFTKRGWAWDGKSVVND-KKKEKA-S-------
sp|P48169|GBRA4_HUMAN   VCFAFVFSALIEFAAVNYFTNIQMEKAKRKTSKP-PQEVPAAPVQREKHP
sp|P62812|GBRA1_MOUSE   VCYAFVFSALIEFATVNYFTKRGYAWDGKSVVPE-KPKKVKDP-------
sp|Q16445|GBRA6_HUMAN   VCFAFVFSALIEFAAVNYFTNLQTQKAKRKAQFA-AP----PTVTISKAT
sp|Q8BHJ7|GBRA5_MOUSE   VCYAFVFSALIEFATVNYFTKRGWAWDGKKALEAAKIKKKEREL------
sp|Q9D6F4|GBRA4_MOUSE   VCFAFVFSALIEFAAVNYFTNIQMQKAKKKISKP-PPEVPAAPVLKEKHT
                        **:***********:*****:       :.
```

```
sp|P14867|GBRA1_HUMAN  ------LIK-KNNTYAPTA-------------------------------
sp|P16305|GBRA6_MOUSE  ESLQAEIVVHSDSKYHLKKRISSL--------------------TLPIVP
sp|P26048|GBRA2_MOUSE  ------VMI-QNNAYAVAV-------------------------------
sp|P26049|GBRA3_MOUSE  ------PTK-KNTTFNIVG-------------------------------
sp|P31644|GBRA5_HUMAN  ------ILNKSTNAFTTGK-------------------------------
sp|P34903|GBRA3_HUMAN  ------PAKKTSTTFNIVG-------------------------------
sp|P47869|GBRA2_HUMAN  ------VMI-QNNAYAVAV-------------------------------
sp|P48169|GBRA4_HUMAN  EA----PLQNTNANLNMRKRTNALVHSESDVGNRTEVGNHSSKSSTVVQE
sp|P62812|GBRA1_MOUSE  ------LIK-KNNTYAPTA-------------------------------
sp|Q16445|GBRA6_HUMAN  EPLEAEIVLHPDSKYHLKKRITSL--------------------SLPIVS
sp|Q8BHJ7|GBRA5_MOUSE  ------ILNKSTNAFTTGK-------------------------------
sp|Q9D6F4|GBRA4_MOUSE  ET----SLQNTHANLNMRKRTNALVHSESDVKSRTEVGNHSSK-TSAVQE

sp|P14867|GBRA1_HUMAN  ---------TSYTPN-LARGDPGLATIAK-SATIEPK-EV----------
sp|P16305|GBRA6_MOUSE  SSEAS-------------------KALSR---------------------
sp|P26048|GBRA2_MOUSE  ---------ANYAPN-LSK-DPVLSTISK-SATTPEP-NK----------
sp|P26049|GBRA3_MOUSE  ---------TTYPIN-LAK-DTEFSTISK-SAAAPSA-SSTP-TAI----
sp|P31644|GBRA5_HUMAN  ---------MSHPPN-IPK-EQTPAGTS----NTTSV-SV----------
sp|P34903|GBRA3_HUMAN  ---------TTYPIN-LAK-DTEFSTISK-GA-APSA-SSTP-TII----
sp|P47869|GBRA2_HUMAN  ---------ANYAPN-LSK-DPVLSTISK-SATTPEP-NK----------
sp|P48169|GBRA4_HUMAN  SSKGTPRSYLASSPNPFSRA-NAAETISA-ARALPSA-SPTSIRTGYMPR
sp|P62812|GBRA1_MOUSE  ---------TSYTPN-LARGDPGLATIAK-SATIEPK-EV----------
sp|Q16445|GBRA6_HUMAN  SSEAN-------------------KVLTR---------------------
sp|Q8BHJ7|GBRA5_MOUSE  ---------LTHPPN-IPK-EQPPAGTA----NAPTV-SI----------
sp|Q9D6F4|GBRA4_MOUSE  SSEATPKAHLASSPNPFSRA-NAAETMSAAARGLSSAASPSP-HGTLRPA
                                                  :

sp|P14867|GBRA1_HUMAN  ----------------------------------KPETKPPEPKKTFNSV
sp|P16305|GBRA6_MOUSE  -----------------------------TPILKSTPVSPPLLLPATGGT
sp|P26048|GBRA2_MOUSE  ----------------------------------KPENKPAEAKKTFNSV
sp|P26049|GBRA3_MOUSE  ----------------------------ASPKATYVQDSPAE-TKTYNSV
sp|P31644|GBRA5_HUMAN  -------------------------------K--PSEEKTSESKKTYNSI
sp|P34903|GBRA3_HUMAN  ----------------------------ASPKATYVQDSPTE-TKTYNSV
sp|P47869|GBRA2_HUMAN  ----------------------------------KPENKPAEAKKTFNSV
sp|P48169|GBRA4_HUMAN  KASVGSASTRHVFGSRLQRIKTTVNTIGATGKLSATP-PPSAPPPSGSGT
sp|P62812|GBRA1_MOUSE  ----------------------------------KPETKPPEPKKTFNSV
sp|Q16445|GBRA6_HUMAN  -----------------------------APILQSTPVTPPPLSPAFGGT
sp|Q8BHJ7|GBRA5_MOUSE  -------------------------------K--ASEEKTAESKKTYNSI
sp|Q9D6F4|GBRA4_MOUSE  S--LGSASTRPAFGSRLGRIKTTVNTTGAAGNVSATP-PPPAPPPSGSGT
                                                              ..    : ..

sp|P14867|GBRA1_HUMAN  SKIDRLSRIAFPLLFGIFNLVYWATYLNREPQLKAPTPHQ
sp|P16305|GBRA6_MOUSE  SKIDQYSRILFPVAFAGFNLVYWIVYLSKDTMEV-SSTVE
sp|P26048|GBRA2_MOUSE  SKIDRMSRIVFPVLFGTFNLVYWATYLNREPVLGV---SP
sp|P26049|GBRA3_MOUSE  SKVDKISRIIFPVLFAIFNLVYWATYVNRESAIKGMIRKQ
sp|P31644|GBRA5_HUMAN  SKIDKMSRIVFPVLFGTFNLVYWATYLNREPVIKGAASPK
sp|P34903|GBRA3_HUMAN  SKVDKISRIIFPVLFAIFNLVYWATYVNRESAIKGMIRKQ
sp|P47869|GBRA2_HUMAN  SKIDRMSRIVFPVLFGTFNLVYWATYLNREPVLGV---SP
sp|P48169|GBRA4_HUMAN  SKIDKYARILFPVTFGAFNMVYWVVYLSKDTMEK-SESLM
sp|P62812|GBRA1_MOUSE  SKIDRLSRIAFPLLFGIFNLVYWATYLNREPQLKAPTPHQ
sp|Q16445|GBRA6_HUMAN  SKIDQYSRILFPVAFAGFNLVYWVVYLSKDTMEV-SSSVE
sp|Q8BHJ7|GBRA5_MOUSE  SKIDKMSRIVFPILFGTFNLVYWATYLNREPVIKGATSPK
sp|Q9D6F4|GBRA4_MOUSE  SKIDKYARILFPVTFGAFNMVYWVVYLSKDTMEK-SESLM
                       **:*: :** **: *. **:*** .*:.::.
```

Supplementary Table 3: ClustalW2 alignment of murine and human α1-6 subunits. Unique amino acid codes are shown. (*) depicts positions that have a single and fully conserved residue; (:) depicts conservation between groups of strongly similar properties with a score greater than 0.5 on the PAM 250 matrix; (.) shows conservation between groups of weakly similar properties with a score less than or equal to 0.5 on the PAM 250 matrix. Yellow and green highlights show the template human α1 and the murine α4 primary sequences, respectively. Red highlights show residues forming the unresolved intracellular loop and not included in the Modeller alignment file. Purple highlights show the alignment of murine α1 and α4 sequences shown in Fig. 3e.

Supplementary Table 4: ClustalW2 alignment of murine and human β1-3 subunits.

```
sp|P18505|GBRB1_HUMAN   MWTVQNRESLGLLSFPVMITMVCCAHSTNEPSNMSYVKETVDRLLKGYDI
sp|P28472|GBRB3_HUMAN   MWGLAGGRLFGIFSAPVLVAVVCCAQSVNDPGNMSFVKETVDKLLKGYDI
sp|P47870|GBRB2_HUMAN   MWRVRKRGYFGIWSFPLIIAAV-CAQSVNDPSNMSLVKETVDRLLKGYDI
sp|P50571|GBRB1_MOUSE   MWTVQNRESLGLLSFPVMVAMVCCAHSSNEPSNMSYVKETVDRLLKGYDI
sp|P63080|GBRB3_MOUSE   MWGFAGGRLFGIFSAPVLVAVVCCAQSVNDPGNMSFVKETVDKLLKGYDI
sp|P63137|GBRB2_MOUSE   MWRVRKRGYFGIWSFPLIIAAV-CAQSVNDPSNMSLVKETVDRLLKGYDI
                        ** .     :*: * *:::: * **:* *:*.*** ******:*******

sp|P18505|GBRB1_HUMAN   RLRPDFGGPPVDVGMRIDVASIDMVSEVNMDYTLTMYFQQSWKDKRLSYS
sp|P28472|GBRB3_HUMAN   RLRPDFGGPPVCVGMNIDIASIDMVSEVNMDYTLTMYFQQYWRDKRLAYS
sp|P47870|GBRB2_HUMAN   RLRPDFGGPPVAVGMNIDIASIDMVSEVNMDYTLTMYFQQAWRDKRLSYN
sp|P50571|GBRB1_MOUSE   RLRPDFGGPPVDVGMRIDVASIDMVSEVNMDYTLTMYFQQSWKDKRLSYS
sp|P63080|GBRB3_MOUSE   RLRPDFGGPPVCVGMNIDIASIDMVSEVNMDYTLTMYFQQYWRDKRLAYS
sp|P63137|GBRB2_MOUSE   RLRPDFGGPPVAVGMNIDIASIDMVSEVNMDYTLTMYFQQAWRDKRLSYN
                        *********** ***.**:********************* *:****:*.

sp|P18505|GBRB1_HUMAN   GIPLNLTLDNRVADQLWVPDTYFLNDKKSFVHGVTVKNRMIRLHPDGTVL
sp|P28472|GBRB3_HUMAN   GIPLNLTLDNRVADQLWVPDTYFLNDKKSFVHGVTVKNRMIRLHPDGTVL
sp|P47870|GBRB2_HUMAN   VIPLNLTLDNRVADQLWVPDTYFLNDKKSFVHGVTVKNRMIRLHPDGTVL
sp|P50571|GBRB1_MOUSE   GIPLNLTLDNRVADQLWVPDTYFLNDKKSFVHGVTVKNRMIRLHPDGTVL
sp|P63080|GBRB3_MOUSE   GIPLNLTLDNRVADQLWVPDTYFLNDKKSFVHGVTVKNRMIRLHPDGTVL
sp|P63137|GBRB2_MOUSE   VIPLNLTLDNRVADQLWVPDTYFLNDKKSFVHGVTVKNRMIRLHPDGTVL
                         *************************************************

sp|P18505|GBRB1_HUMAN   YGLRITTTAACMMDLRRYPLDEQNCTLEIESYGYTTDDIEFYWNGGEGAV
sp|P28472|GBRB3_HUMAN   YGLRITTTAACMMDLRRYPLDEQNCTLEIESYGYTTDDIEFYWRGGDKAV
sp|P47870|GBRB2_HUMAN   YGLRITTTAACMMDLRRYPLDEQNCTLEIESYGYTTDDIEFYWRGDDNAV
sp|P50571|GBRB1_MOUSE   YGLRITTTAACMMDLRRYPLDEQNCTLEIESYGYTTDDIEFYWNGGEGAV
sp|P63080|GBRB3_MOUSE   YGLRITTTAACMMDLRRYPLDEQNCTLEIESYGYTTDDIEFYWRGGDKAV
sp|P63137|GBRB2_MOUSE   YGLRITTTAACMMDLRRYPLDEQNCTLEIESYGYTTDDIEFYWRGDDNAV
                        *******************************************.*.: **

sp|P18505|GBRB1_HUMAN   TGVNKIELPQFSIVDYKMVSKKVEFTTGAYPRLSLSFRLKRNIGYFILQT
sp|P28472|GBRB3_HUMAN   TGVERIELPQFSIVEHRLVSRNVVFATGAYPRLSLSFRLKRNIGYFILQT
sp|P47870|GBRB2_HUMAN   TGVTKIELPQFSIVDYKLITKKVVFSTGSYPRLSLSFKLKRNIGYFILQT
sp|P50571|GBRB1_MOUSE   TGVNKIELPQFSIVDYKMVSKKVEFTTGAYPRLSLSFRLKRNIGYFILQT
sp|P63080|GBRB3_MOUSE   TGVERIELPQFSIVEHRLVSRNVVFATGAYPRLSLSFRLKRNIGYFILQT
sp|P63137|GBRB2_MOUSE   TGVTKIELPQFSIVDYKLITKKVVFSTGSYPRLSLSFKLKRNIGYFILQT
                        *** :*********::::::::* *:**:********:************

sp|P18505|GBRB1_HUMAN   YMPSTLITILSWVSFWINYDASAARVALGITTVLTMTTISTHLRETLPKI
sp|P28472|GBRB3_HUMAN   YMPSILITILSWVSFWINYDASAARVALGITTVLTMTTINTHLRETLPKI
sp|P47870|GBRB2_HUMAN   YMPSILITILSWVSFWINYDASAARVALGITTVLTMTTINTHLRETLPKI
sp|P50571|GBRB1_MOUSE   YMPSTLITILSWVSFWINYDASAARVALGITTVLTMTTISTHLRETLPKI
sp|P63080|GBRB3_MOUSE   YMPSILITILSWVSFWINYDASAARVALGITTVLTMTTINTHLRETLPKI
sp|P63137|GBRB2_MOUSE   YMPSILITILSWVSFWINYDASAARVALGITTVLTMTTINTHLRETLPKI
                        **** **********************************.**********

sp|P18505|GBRB1_HUMAN   PYVKAIDIYLMGCFVFVFLALLEYAFVNYIFFGKGPQK--KGASKQDQSA
sp|P28472|GBRB3_HUMAN   PYVKAIDMYLMGCFVFVFLALLEYAFVNYIFFGRGPQRQKKLAEKTAKAK
sp|P47870|GBRB2_HUMAN   PYVKAIDMYLMGCFVFVFMALLEYALVNYIFFGRGPQRQKKAAEKAASAN
sp|P50571|GBRB1_MOUSE   PYVKAIDIYLMGCFVFVFLALLEYAFVNYIFFGKGPQK--KGASKQDQSA
sp|P63080|GBRB3_MOUSE   PYVKAIDMYLMGCFVFVFLALLEYAFVNYIFFGRGPQRQKKLAEKTAKAK
sp|P63137|GBRB2_MOUSE   PYVKAIDMYLMGCFVFVFMALLEYALVNYIFFGRGPQRQKKAAEKAANAN
                        *******:**********:******:*******:***:  * *.*   .:
```

```
sp|P18505|GBRB1_HUMAN  NEKNKLEMNKV---------------------------------------QVD
sp|P28472|GBRB3_HUMAN  NDRSKSESNR-----------------------------------------VD
sp|P47870|GBRB2_HUMAN  NEKMRLDVNKIFYKDIKQNGTQYRSLWDPTGNLSPTRRTTNYDFSLYTMD
sp|P50571|GBRB1_MOUSE  NEKNRLEMNKV---------------------------------------QVD
sp|P63080|GBRB3_MOUSE  NDRSKSEINR-----------------------------------------VD
sp|P63137|GBRB2_MOUSE  NEKMRLDVNKMFYKDIKQNGTQYRSLWDPTGDLSPTRRTTNYDFSLYTMD
                       *:: : : *:                                      :*

sp|P18505|GBRB1_HUMAN  AHGNILLSTLEIRNETSGSEVLTSVSDPKATMYSYDSASIQYRKPLSSRE
sp|P28472|GBRB3_HUMAN  AHGNILLTSLEVHNEMN--EVSGGIGDTRNSAISFDNSGIQYRKQSMPRE
sp|P47870|GBRB2_HUMAN  PHENILLSTLEIKNEMATSEAVMGLGDPRSTMLAYDASSIQYRKAGLPRH
sp|P50571|GBRB1_MOUSE  AHGNILLSTLEIRNETSGSEVLTGVSDPKATMYSYDSASIQYRKPLSSRE
sp|P63080|GBRB3_MOUSE  AHGNILLAPMDVHNEMN--EVAGSVGDTRNSAISFDNSGIQYRKQSMPKE
sp|P63137|GBRB2_MOUSE  PHENILLSTLEIKNEMATSEAVMGLGDPRSTMLAYDASSIQYRKAGLPRH
                       .* ****:.::::**    *.  .:.*.: :  ::* :.*****   .:.

sp|P18505|GBRB1_HUMAN  AYGRAL-DRHGVPSKGRIRRRASQLKVKIPDLTDVNSIDKWSRMFFPITF
sp|P28472|GBRB3_HUMAN  GHGRFLGDRSLPHKKTHLRRRSSQLKIKIPDLTDVNAIDRWSRIVFPFTF
sp|P47870|GBRB2_HUMAN  SFGRNALERHVAQKKSRLRRRASQLKITIPDLTDVNAIDRWSRIFFPVVF
sp|P50571|GBRB1_MOUSE  GFGRGL-DRHGVPGKGRIRRRASQLKVKIPDLTDVNSIDKWSRMFFPITF
sp|P63080|GBRB3_MOUSE  GHGRYMGDRSIPHKKTHLRRRSSQLKIKIPDLTDVNAIDRWSRIVFPFTF
sp|P63137|GBRB2_MOUSE  SFGRNALERHVAQKKSRLRRRASQLKITIPDLTDVNAIDRWSRIFFPVVF
                       ..**   :*     * ::***:****:.********:**:***:.**..*

sp|P18505|GBRB1_HUMAN  SLFNVVYWLYYVH
sp|P28472|GBRB3_HUMAN  SLFNLVYWLYYVN
sp|P47870|GBRB2_HUMAN  SFFNIVYWLYYVN
sp|P50571|GBRB1_MOUSE  SLFNVVYWLYYVH
sp|P63080|GBRB3_MOUSE  SLFNLVYWLYYVN
sp|P63137|GBRB2_MOUSE  SFFNIVYWLYYVN
                       *:**:*******:
```

Supplementary Table 4: ClustalW2 alignment of murine and human β1-3 subunits. See Supplementary Table 3 legend for identification of the codes (*), (:), (.), and highlights. Purple highlights show the alignment of murine β2 and β3 sequences shown in Fig. 2c.

Supplementary Table 5: ClustalW2 alignment of murine and human γ1-3 (GBRG1-3), δ (GBRD), ε (GBRE), θ (GBRT), π (GBRP).

```
sp|O00591|GBRP_HUMAN   MNYSL--------------------HLAFVCLSLFTE-RM-CI-QG----
sp|O14764|GBRD_HUMAN   MDAPA--------------------RLLAPLLLLCAQ-QLRGTRAM----
sp|P18507|GBRG2_HUMAN  MSSPNIWSTGSSVYSTPVFSQKMT-VWILLLLSLYPGF--TSQKSD----
sp|P22723|GBRG2_MOUSE  MSSPNTWSIGSSVY-SPVFSQKMT-LWILLLLSLYPGF--TSQKSD----
sp|P22933|GBRD_MOUSE   MDVLG--------------------WLLLPLLLLCTQ-PHHGARAM----
sp|P27681|GBRG3_MOUSE  MAAK-----------------------LLLLLCLFSGLHARSRRVE----
sp|P78334|GBRE_HUMAN   MLSKV--------------------LPVLLGILLIL--QSRVEGPQ----
sp|Q8N1C3|GBRG1_HUMAN  MGPLKAFLFSPF----LLRSQSRGVRLVFLLLTLHLGN--CVDKAD----
sp|Q8QZW7|GBRP_MOUSE   MSYSL--------------------YLAFLCLSLLTQ-RT-CI-QG----
sp|Q99928|GBRG3_HUMAN  MAPK-----------------------LLLLLCLFSGLHARSRKVE----
sp|Q9JLF1|GBRT_MOUSE   MGIRG--------------------MLRAAALLLLIR-TWLAESNGPSPT
sp|Q9R0Y8|GBRG1_MOUSE  MGSGKAFLFSPS----LLWSQTRGVRLIFLLLTLHLGN--CVDKAD----
sp|Q9UN88|GBRT_HUMAN   MGIRG--------------------MLRAAVILLLIR-TWLAEGNYPSPI
                       *                              : *

sp|O00591|GBRP_HUMAN   SQFNVE-VG-----------------------------------RSDKL-
sp|O14764|GBRD_HUMAN   NDIGDY-VG-----------------------------------SNLEIS
sp|P18507|GBRG2_HUMAN  -DD-YEDYASNKTWVLTPKVP--------------------------EGD
sp|P22723|GBRG2_MOUSE  -DD-YEDYASNKTWVLTPKVP--------------------------EGD
sp|P22933|GBRD_MOUSE   NDIGDY-VG-----------------------------------SNLEIS
sp|P27681|GBRG3_MOUSE  EDE-NEDSPSNQKWVLAPKSQ--------------------------DTD
sp|P78334|GBRE_HUMAN   TE--SKNEASSRDVVYGPQPQPLENQLLSEETKSTETETGSRVGK--LPE
sp|Q8N1C3|GBRG1_HUMAN  DED-DEDLTVNKTWVLAPKIH--------------------------EGD
sp|Q8QZW7|GBRP_MOUSE   NQVNVE-VS-----------------------------------RSDKL-
sp|Q99928|GBRG3_HUMAN  EDE-YEDSSSNQKWVLAPKSQ--------------------------DTD
sp|Q9JLF1|GBRT_MOUSE   PKFHFELSSSTPEVILD---------------------LFNCKNCANEAV
sp|Q9R0Y8|GBRG1_MOUSE  DED-DEDLTMNKTWVLAPKIH--------------------------EGD
sp|Q9UN88|GBRT_HUMAN   PKFHFEFSSAVPEVVLN---------------------LFNCKNCANEAV
                   .

sp|O00591|GBRP_HUMAN   SLPGFENLTAGYNKFLRPNFGGEPVQIALTLDIASISSISESNMDYTATI
sp|O14764|GBRD_HUMAN   WLPNLDGLIAGYARNFRPGIGGPPVNVALALEVASIDHISEANMEYTMTV
sp|P18507|GBRG2_HUMAN  VTVILNNLLEGYDNKLRPDIGVKPTLIHTDMYVNSIGPVNAINMEYTIDI
sp|P22723|GBRG2_MOUSE  VTVILNNLLEGYDNKLRPDIGVKPTLIHTDMYVNSIGPVNAINMEYTIDI
sp|P22933|GBRD_MOUSE   WLPNLDGLMEGYARNFRPGIGGAPVNVALALEVASIDHISEANMEYTMTV
sp|P27681|GBRG3_MOUSE  VTLILNKLLREYDKKLRPDIGIKPTVIDVDIYVNSIGPVSSINMEYQIDI
sp|P78334|GBRE_HUMAN   ASRILNTILSNYDHKLRPGIGEKPTVVTVEISVNSLGPLSILDMEYTIDI
sp|Q8N1C3|GBRG1_HUMAN  ITQILNSLLQGYDNKLRPDIGVRPTVIETDVYVNSIGPVDPINMEYTIDI
sp|Q8QZW7|GBRP_MOUSE   SLPGFENLTAGYNKFLRPNFGGDPVRIALTLDIASISSISESNMDYTATI
sp|Q99928|GBRG3_HUMAN  VTLILNKLLREYDKKLRPDIGIKPTVIDVDIYVNSIGPVSSINMEYQIDI
sp|Q9JLF1|GBRT_MOUSE   VQKILDRVLSTYDVRLRPNFGGAPVPVSVSIYVSSIEQISEINMDYTITM
sp|Q9R0Y8|GBRG1_MOUSE  ITQILNSLLQGYDNKLRPDIGVRPTVIETDVYVNSIGPVDPINMEYTIDI
sp|Q9UN88|GBRT_HUMAN   VQKILDRVLSRYDVRLRPNFGGAPVPVRISIYVTSIEQISEMNMDYTITM
                           :: :   *   :**.:*  *. :   : : *:  :.  :*:*   :

sp|O00591|GBRP_HUMAN   YLRQRWMDQRLVFEG-NKSFTLDARLVEFLWVPDTYIVESKKSFLHEVTV
sp|O14764|GBRD_HUMAN   FLHQSWRDSRLSYNHTNETLGLDSRFVDKLWLPDTFIVNAKSAWFHDVTV
sp|P18507|GBRG2_HUMAN  FFAQTWYDRRLKFNSTIKVLRLNSNMVGKIWIPDTFFRNSKKADAHWITT
sp|P22723|GBRG2_MOUSE  FFAQTWYDRRLKFNSTIKVLRLNSNMVGKIWIPDTFFRNSKKADAHWITT
sp|P22933|GBRD_MOUSE   FLHQSWRDSRLSYNHTNETLGLDSRFVDKLWLPDTFIVNAKSAWFHDVTV
sp|P27681|GBRG3_MOUSE  FFAQTWTDSRLRFNSTMKILTLNSNMVGLIWIPDTIFRNSKTAEAHWITT
sp|P78334|GBRE_HUMAN   IFSQTWYDERLCYNDTFESLVLNGNVVSQLWIPDTFFRNSKRTHEHEITM
sp|Q8N1C3|GBRG1_HUMAN  IFAQTWFDSRLKFNSTMKVLMLNSNMVGKIWIPDTFFRNSRKSDAHWITT
sp|Q8QZW7|GBRP_MOUSE   YLRQRWTDPRLVFEG-NKSFTLDARLVEFLWVPDTYIVESKKSFLHEVTV
sp|Q99928|GBRG3_HUMAN  FFAQTWTDSRLRFNSTMKILTLNSNMVGLIWIPDTIFRNSKTAEAHWITT
sp|Q9JLF1|GBRT_MOUSE   FLHQTWKDTRLAYYETNLNLTLDYRMHEKLWVPDCYFVNSKDAFVHDVTV
sp|Q9R0Y8|GBRG1_MOUSE  IFAQTWFDSRLKFNSTMKVLMLNSNMVGKIWIPDTFFRNSRKSDAHWITT
sp|Q9UN88|GBRT_HUMAN   FFHQTWKDSRLAYYETTLNLTLDYRMHEKLWVPDCYFLNSKDAFVHDVTV
```

```
                         : * * * ** :      : *: ..   :*:**  : ::: :  * :*
sp|O00591|GBRP_HUMAN    GNRLIRLFSNGTVLYALRITTTVACNMDLSKYPMDTQTCKLQLESWGYDG
sp|O14764|GBRD_HUMAN    ENKLIRLQPDGVILYSIRITSTVACDMDLAKYPMDEQECMLDLESYGYSS
sp|P18507|GBRG2_HUMAN   PNRMLRIWNDGRVLYTLRLTIDAECQLQLHNFPMDEHSCPLEFSSYGYPR
sp|P22723|GBRG2_MOUSE   PNRMLRIWNDGRVLYTLRLTIDAECQLQLHNFPMDEHSCPLEFSSYGYPR
sp|P22933|GBRD_MOUSE    ENKLIRLQPDGVILYSIRITSTVACDMDLAKYPLDEQECMLDLESYGYSS
sp|P27681|GBRG3_MOUSE   PNQLLRIWNDGKILYTLRLTINAECQLQLHNFPMDAHACPLTFSSYGYPK
sp|P78334|GBRE_HUMAN    PNQMVRIYKDGKVLYTIRMTIDAGCSLHMLRFPMDSHSCPLSFSSFSYPE
sp|Q8N1C3|GBRG1_HUMAN   PNRLLRIWNDGRVLYTLRLTINAECYLQLHNFPMDEHSCPLEFSSYGYPK
sp|Q8QZW7|GBRP_MOUSE    GNRLIRLFSNGTVLYALRITTTVTCNMDLSKYPMDTQTCKLQLESWGYDG
sp|Q99928|GBRG3_HUMAN   PNQLLRIWNDGKILYTLRLTINAECQLQLHNFPMDEHSCPLIFSSYGYPK
sp|Q9JLF1|GBRT_MOUSE    ENRVFQLHPDGTVRYGIRLTTTAACSLDLQKFPMDKQSCKLEVESYGYTV
sp|Q9R0Y8|GBRG1_MOUSE   PNRLLRIWSDGRVLYTLRLTINAECYLQLHNFPMDEHSCPLEFSSYGYPK
sp|Q9UN88|GBRT_HUMAN    ENRVFQLHPDGTVRYGIRLTTTAACSLDLHKFPMDKQACNLVVESYGYTV
                         *::.::  :* : * :*:*  . * :.: .:*:* : * * ..*:.*

sp|O00591|GBRP_HUMAN    NDVEFTWLRGNDSVRGLEHLRLAQYTIERYFTLVTRSQ-QETGNYTRLVL
sp|O14764|GBRD_HUMAN    EDIVYYWSESQEHIHGLDKLQLAQFTITSYRFTTELMNFKSAGQFPRLSL
sp|P18507|GBRG2_HUMAN   EEIVYQWKRSSVEVGDTRSWRLYQFSFVGLRNTTEVVK-TTSGDYVVMSV
sp|P22723|GBRG2_MOUSE   EEIVYQWKRSSVEVGDTRSWRLYQFSFVGLRNTTEVVK-TTSGDYVVMSV
sp|P22933|GBRD_MOUSE    EDIVYYWSENQEQIHGLDRLQLAQFTITSYRFTTELMNFKSAGQFPRLSL
sp|P27681|GBRG3_MOUSE   EEMIYRWRKNSVEAADQKSWRLYQFDFMGLRNTTEIVT-TSAGDYVVMTI
sp|P78334|GBRE_HUMAN    NEMIYKWENFKLEINEKNSWKLFQFDFTGVSNKTEIIT-TPVGDFMVMTI
sp|Q8N1C3|GBRG1_HUMAN   NEIEYKWKKPSVEVADPKYWRLYQFAFVGLRNSTEITH-TISGDYVIMTI
sp|Q8QZW7|GBRP_MOUSE    NDVEFSWLRGNDSVRGLENLRLAQYTIQQYFTLVTVSQ-QETGNYTRLVL
sp|Q99928|GBRG3_HUMAN   EEMIYRWRKNSVEAADQKSWRLYQFDFMGLRNTTEIVT-TSAGDYVVMTI
sp|Q9JLF1|GBRT_MOUSE    EDIVLSWEDD-NAIHITDGLHIPQYTYLGRTITSKEVY-FYTGSYMRLIV
sp|Q9R0Y8|GBRG1_MOUSE   NEIEYKWKKPSVEVADPKYWRLYQFAFVGLRNSTEISH-TISGDYIIMTI
sp|Q9UN88|GBRT_HUMAN    EDIILFWDDNGNAIHMTEELHIPQFTFLGRTITSKEVY-FYTGSYIRLIL
                        :::   *             :: *:                 *.:  :  :

sp|O00591|GBRP_HUMAN    QFELRRNVLYFILETYVPSTFLVVLSWVSFWISLDSVPARTCIGVTTVLS
sp|O14764|GBRD_HUMAN    HFHLRRNRGVYIIQSYMPSVLLVAMSWVSFWISQAAVPARVSLGITTVLT
sp|P18507|GBRG2_HUMAN   YFDLSRRMGYFTIQTYIPCTLIVVLSWVSFWINKDAVPARTSLGITTVLT
sp|P22723|GBRG2_MOUSE   YFDLSRRMGYFTIQTYIPCTLIVVLSWVSFWINKDAVPARTSLGITTVLT
sp|P22933|GBRD_MOUSE    HFQLRRNRGVYIIQSYMPSVLLVAMSWVSFWISQAAVPARVSLGITTVLT
sp|P27681|GBRG3_MOUSE   YFELSRRMGYFTIQTYIPCILTVVLSWVSFWIKKDATPARTTLGITTVLT
sp|P78334|GBRE_HUMAN    FFNVSRRFGYVAFQNYVPSSVTTMLSWVSFWIKTESAPARTSLGITSVLT
sp|Q8N1C3|GBRG1_HUMAN   FFDLSRRMGYFTIQTYIPCILTVVLSWVSFWINKDAVPARTSLGITTVLT
sp|Q8QZW7|GBRP_MOUSE    QFELRRNVLYFILETYVPSTFLVVLSWVSFWISLDSVPARTCIGVTTVLS
sp|Q99928|GBRG3_HUMAN   YFELSRRMGYFTIQTYIPCILTVVLSWVSFWIKKDATPARTALGITTVLT
sp|Q9JLF1|GBRT_MOUSE    KFQVQREVRSYLVQVYWPTVLTTILSWISFWMNYDSSAARVTIGLTSILV
sp|Q9R0Y8|GBRG1_MOUSE   FFDLSRRMGYFTIQTYIPCILTVVLSWVSFWINKDAVPARTSLGITTVLT
sp|Q9UN88|GBRT_HUMAN    KFQVQREVNSYLVQVYWPTVLTTITSWISFWMNYDSSAARVTIGLTSMLI
                         *.: *.     .: * *  . .  **:***:.  : .**. :*:*::*

sp|O00591|GBRP_HUMAN    MTTLMIGSRTSLPNTNCFIKAIDVYLGICFSFVFGALLEYAVAHYSSLQQ
sp|O14764|GBRD_HUMAN    MTTLMVSARSSLPRAS-AIKALDVYFWICYVFVFAALVEYAFAHFNADYR
sp|P18507|GBRG2_HUMAN   MTTLSTIARKSLPKVS-YVTAMDLFVSVCFIFVFSALVEYGTLHYFVSNR
sp|P22723|GBRG2_MOUSE   MTTLSTIARKSLPKVS-YVTAMDLFVSVCFIFVFSALVEYGTLHYFVSNR
sp|P22933|GBRD_MOUSE    MTTLMVSARSSLPRAS-AIKALDVYFWICYVFVFAALVEYAFAHFNADYR
sp|P27681|GBRG3_MOUSE   MTTLSTIARKSLPRVS-YVTAMDLFVTVCFLFVFAALMEYATLNYYSSCR
sp|P78334|GBRE_HUMAN    MTTLGTFSRKNFPRVS-YITALDFYIAICFVFCFCALLEFAVLNFLIYNQ
sp|Q8N1C3|GBRG1_HUMAN   MTTLSTIARKSLPKVS-YVTAMDLFVSVCFIFVFAALMEYGTLHYFTSNQ
sp|Q8QZW7|GBRP_MOUSE    MTTLMIGSRTSLPNTNCFIKAIDVYLGICFSFVFGALLEYAVAHYSSLQQ
sp|Q99928|GBRG3_HUMAN   MTTLSTIARKSLPRVS-YVTAMDLFVTVCFLFVFAALMEYATLNYYSSCR
sp|Q9JLF1|GBRT_MOUSE    LTTIDSHMRDKLPHIS-CIKAIDIYILVCLFFVFLSLLEYVYINYLFFSQ
sp|Q9R0Y8|GBRG1_MOUSE   MTTLSTIARKSLPKVS-YVTAMDLFVSVCFIFVFAALMEYGTLHYFTSNN
sp|Q9UN88|GBRT_HUMAN    LTTIDSHLRDKLPNIS-CIKAIDIYILVCLFFVFLSLLEYVYINYLFYSR
                        :**:    * .:*. .  :.*:*.:. :*  * * :*:*:   ::    .
```

```
sp|O00591|GBRP_HUMAN    MAAK-DRGTTK--E-VEEVS------------------------------
sp|O14764|GBRD_HUMAN    KKQK-AKVKVS--RPRAEMD------------------------------
sp|P18507|GBRG2_HUMAN   KPSK-DKDKKK-KNPA----------------------------------
sp|P22723|GBRG2_MOUSE   KPSK-DKDKKK-KNPLLR--------------------------------
sp|P22933|GBRD_MOUSE    KKRK-AKVKVT--KPRAEMD------------------------------
sp|P27681|GBRG3_MOUSE   KPTI-RKKKTSLLHPDSTRW------------------------------
sp|P78334|GBRE_HUMAN    TKAH-ASPKL--RHPRIN--------------------------------
sp|Q8N1C3|GBRG1_HUMAN   KGKTATKDRKL-KNKA----------------------------------
sp|Q8QZW7|GBRP_MOUSE    MAVK-DRGPAK--D-SEEVN------------------------------
sp|Q99928|GBRG3_HUMAN   KPTT-TKKTTSLLHPDSSRW------------------------------
sp|Q9JLF1|GBRT_MOUSE    VPRR-NHRRCR--KPRRVVARYRYQEVVVANVQDGLINVEDRVEDRAGPL
sp|Q9R0Y8|GBRG1_MOUSE   KGKT-TRGRKL-KNKT----------------------------------
sp|Q9UN88|GBRT_HUMAN    GPRR-QPRRHR--RPRRVIARYRYQQVVVGNVQDGLINVEDGV----SSL

sp|O00591|GBRP_HUMAN    --------------------------ITNIIN---S----S--ISS-FKR
sp|O14764|GBRD_HUMAN    --------------------------VRN-----------A--IV-----
sp|P18507|GBRG2_HUMAN   -----------------------------------P----TIDIRP-RSA
sp|P22723|GBRG2_MOUSE   -----------------------------MFSFKAP----TIDIRP-RSA
sp|P22933|GBRD_MOUSE    --------------------------VRN-----------A--IV-----
sp|P27681|GBRG3_MOUSE   --------------------------IPDRISLQAPSNYSLLDMRPPPPV
sp|P78334|GBRE_HUMAN    -----------------------------------------SRAHA-RT-
sp|Q8N1C3|GBRG1_HUMAN   -------------------------------S---M----TPGLHP-GST
sp|Q8QZW7|GBRP_MOUSE    --------------------------ITNIIN---S----S--ISS-FKR
sp|Q99928|GBRG3_HUMAN   --------------------------IPERISLQAPSNYSLLDMRPPPTA
sp|Q9JLF1|GBRT_MOUSE    PDSPMQAHLASQESLGSLVFTSEQAQLATSES---L---SL--LSS-ASS
sp|Q9R0Y8|GBRG1_MOUSE   -------------------------------S---A----SPGLHA-GST
sp|Q9UN88|GBRT_HUMAN    PITPAQAPLASPESLGSLTSTSEQAQLATSES---L---SP--LTS-LSG

sp|O00591|GBRP_HUMAN    KISFASIEIS----------S-----------------------------
sp|O14764|GBRD_HUMAN    LFSLSAAGVT---QEL----A-----------------------------
sp|P18507|GBRG2_HUMAN   TIQMNNATHL---QER----D-----------------------------
sp|P22723|GBRG2_MOUSE   TIQMNNATHL---QER----D-----------------------------
sp|P22933|GBRD_MOUSE    LFSLSAAGVS---QEL----A-----------------------------
sp|P27681|GBRG3_MOUSE   MITLNNSMYW---QEF----E-----------------------------
sp|P78334|GBRE_HUMAN    -RARSRACAR---QHQ----EAFVCQIVTTEGSDGEERPSCSAQQPPSPG
sp|Q8N1C3|GBRG1_HUMAN   LIPMNNISVP---Q------E-----------------------------
sp|Q8QZW7|GBRP_MOUSE    KISFASIEIS----------G-----------------------------
sp|Q99928|GBRG3_HUMAN   MITLNNSVYW---QEF----E-----------------------------
sp|Q9JLF1|GBRT_MOUSE    QTQLATGESLSDLPSTSEQTV-----------------------------
sp|Q9R0Y8|GBRG1_MOUSE   LIPMNSISLP---Q-G----E-----------------------------
sp|Q9UN88|GBRT_HUMAN    QAPLATGESLSDLPSTSEQAR-----------------------------

sp|O00591|GBRP_HUMAN    DNV--------------------------DYS------DLT-MKTSDKFK
sp|O14764|GBRD_HUMAN    ISR--------------------------RQR------RVP-GNLMGSYR
sp|P18507|GBRG2_HUMAN   EEY--------------------------GYE------CLD-GKDCASFF
sp|P22723|GBRG2_MOUSE   EEY--------------------------GYE------CLD-GKDCASFF
sp|P22933|GBRD_MOUSE    ISR--------------------------RQG------RVP-GNLMGSYR
sp|P27681|GBRG3_MOUSE   DTC--------------------------VYE------CLD-GKDCQSFF
sp|P78334|GBRE_HUMAN    SPEGPRSLCS-------------------KLA------CCEWCKRFKKYF
sp|Q8N1C3|GBRG1_HUMAN   DDY--------------------------GYQ------CLE-GKDCASFF
sp|Q8QZW7|GBRP_MOUSE    DNV--------------------------NYS------DLT-MKASDKFK
sp|Q99928|GBRG3_HUMAN   DTC--------------------------VYE------CLD-GKDCQSFF
sp|Q9JLF1|GBRT_MOUSE    PEC------TIHFHGFLTNDSIIPIKIHSRSD------ACD-DEDSEESL
sp|Q9R0Y8|GBRG1_MOUSE   DDY--------------------------GYQ------CLE-GKDCTSFF
sp|Q9UN88|GBRT_HUMAN    HSY------GVRFNGFQADDSIFPTEIRNRVEAHGHGVTHD-HEDSNESL
                                                                   :   .
```

```
sp|O00591|GBRP_HUMAN    FVF------------RE---------------------------------
sp|O14764|GBRD_HUMAN    SVGVETGETKKEGAARSG--------------------------------
sp|P18507|GBRG2_HUMAN   CCF----EDCRTGAWRH---------------------------------
sp|P22723|GBRG2_MOUSE   CCF----EDCRTGAWRH---------------------------------
sp|P22933|GBRD_MOUSE    SVEVE---AKKEGGSRPG--------------------------------
sp|P27681|GBRG3_MOUSE   CCY----EECKSGSWRR---------------------------------
sp|P78334|GBRE_HUMAN    CMV----PDCEGSTWQQ---------------------------------
sp|Q8N1C3|GBRG1_HUMAN   CCF----EDCRTGSWRE---------------------------------
sp|Q8QZW7|GBRP_MOUSE    FVF------------RE---------------------------------
sp|Q99928|GBRG3_HUMAN   CCY----EECKSGSWRK---------------------------------
sp|Q9JLF1|GBRT_MOUSE    S----SEESHGHGSSHTGRLKLQISQRCVQEASWDLDKIEILQDDISITS
sp|Q9R0Y8|GBRG1_MOUSE   CCF----DDCRTGSWRE---------------------------------
sp|Q9UN88|GBRT_HUMAN    S----SDERHGHGP--SGKPMLHHGEKGVQEAGWDL------DDNN-DKS


sp|O00591|GBRP_HUMAN    -------------------------------------------------K
sp|O14764|GBRD_HUMAN    -------------------------------------------------G
sp|P18507|GBRG2_HUMAN   --------------------------------------------------
sp|P22723|GBRG2_MOUSE   --------------------------------------------------
sp|P22933|GBRD_MOUSE    -------------------------------------------------G
sp|P27681|GBRG3_MOUSE   --------------------------------------------------
sp|P78334|GBRE_HUMAN    --------------------------------------------------
sp|Q8N1C3|GBRG1_HUMAN   --------------------------------------------------
sp|Q8QZW7|GBRP_MOUSE    -------------------------------------------------K
sp|Q99928|GBRG3_HUMAN   --------------------------------------------------
sp|Q9JLF1|GBRT_MOUSE    SWLGLDEQCKGDADSIWSLTDEELMACDQEKDSSSESEENCSPSPGCSFN
sp|Q9R0Y8|GBRG1_MOUSE   --------------------------------------------------
sp|Q9UN88|GBRT_HUMAN    DCLAIKEQFKCDTNSTWGLNDDELMAHGQEKDSSSESEDSCPPSPGCSFT


sp|O00591|GBRP_HUMAN    MGRIVDYFTIQNPSNVDHYSKLLFPLIFMLANVFYWAYYMYF
sp|O14764|GBRD_HUMAN    QGGIRARLRPIDADTIDIYARAVFPAAFAAVNVIYWAAYA-M
sp|P18507|GBRG2_HUMAN   -GR-----IHIRIAKMDSYARIFFPTAFCLFNLVYWVSYLYL
sp|P22723|GBRG2_MOUSE   -GR-----IHIRIAKMDSYARIFFPTAFCLFNLVYWVSYLYL
sp|P22933|GBRD_MOUSE    PGGIRSRLKPIDADTIDIYARAVFPAAFAAVNIIYWAAYT-M
sp|P27681|GBRG3_MOUSE   -GR-----IHIDVSELDSYSRVFFPTSFLLFNLVYWVGYLYL
sp|P78334|GBRE_HUMAN    -GR-----LCIHVYRLDNYSRVVFPVTFFFFNVLYWLVCLNL
sp|Q8N1C3|GBRG1_HUMAN   -GR-----IHIRIAKIDSYSRIFFPTAFALFNLVYWVGYLYL
sp|Q8QZW7|GBRP_MOUSE    ISRIIDYFTIQNPSNVDRYSKLLFPLIFMLANVFYWAYYMYF
sp|Q99928|GBRG3_HUMAN   -GR-----IHIDILELDSYSRVFFPTSFLLFNLVYWVGYLYL
sp|Q9JLF1|GBRT_MOUSE    EGFSFQLFKPNRVPKVDRWSRFLFPLSFGLFNVVYWLYHV-Y
sp|Q9R0Y8|GBRG1_MOUSE   -GR-----IHIRIAKIDSYSRIFFPTAFALFNLVYWVGYLYL
sp|Q9UN88|GBRT_HUMAN    EGFSFDLFNPDYVPKVDKWSRFLFPLAFGLFNIVYWVYHM-Y
                         .             :* ::: .**  *   *:.**
```

Supplementary Table 5: ClustalW2 alignment of murine and human γ1-3 (GBRG1-3), δ (GBRD), ε (GBRE), θ (GBRT), π (GBRP). See Supplementary Table 3 legend for identification of the codes (*), (:), (.), and highlights. Purple highlights show the alignment of murine γ2 and δ sequences shown in Fig. 3d.
